# Oleylammonium fluoride passivated blue-emitting 2D CsPbBr_3_ nanoplates with near-unity photoluminescence quantum yield: safeguarding against threats from external perturbations†

**DOI:** 10.1039/d4sc05565a

**Published:** 2024-11-26

**Authors:** Arghya Sen, Abhijit Dutta, Abir Lal Bose, Pratik Sen

**Affiliations:** a Department of Chemistry, Indian Institute of Technology Kanpur Kanpur – 208 016 UP India psen@iitk.ac.in +91 512 259 6806 +91 512 259 6312; b Department of Chemical Engineering, Indian Institute of Technology Kanpur Kanpur – 208 016 UP India

## Abstract

Quantum-confined, two-dimensional (2D) CsPbBr_3_ (CPB) nanoplates (NPLs) have emerged as exceptional candidates for next-generation blue LEDs and display technology applications. However, their large surface-to-volume ratio and detrimental bromide vacancies adversely affect their photoluminescence quantum yield (PLQY). Additionally, external perturbations such as heat, light exposure, moisture, oxygen, and solvent polarity accelerate their transformation into three-dimensional (3D), green-emitting CPB nanocrystals (NCs), thereby resulting in the loss of their quantum confinement. Until now, no reported strategies have successfully addressed all these issues simultaneously. In this study, for the first time, we prepared oleylammonium fluoride (OAmF) salt and applied it post-synthetically to CPB NPLs with thicknesses of *n* = 3 and *n* = 4. Steady state and time-resolved photoluminescence (TRPL) measurements like fluorescence upconversion and TCSPC confirmed the elimination of detrimental deep trap states by fluoride ions, resulting in an unprecedented improvement in PLQY to 85% for *n* = 3 and 98% for *n* = 4. Furthermore, the formation of robust Pb–F bonds, coupled with strong electrostatic and hydrogen-bonding interactions, resulted in a highly stable NPL surface–ligand interaction. This concrete surface architecture restricts the undesired phase transition of 2D NPLs into 3D NCs under various external perturbations, including heat up to 363 K, strong UV irradiation, water, atmospheric conditions, and solvent polarity. Also, the temperature dependent TRPL measurements provide an insight into the charge carrier dynamics under thermal stress conditions and reveal the location of shallow trap states, which lie below 7 meV from the conduction band edge. In brief, our innovative OAmF salt has effectively addressed all the critical issues of 2D CPB NPLs, paving the way for next-generation LED applications. This breakthrough not only enhances the stability and PLQY of CPB NPLs but also offers a scalable solution for the advancement of perovskite-based technologies.

## Abstract 2

Post-synthetic treatment of two-dimensional (2D) colloidal CsPbBr_3_ nanoplates by oleylammonium fluoride has boosted the PLQY upto near unity with significant enhancement of stability from the external exposures like heat, UV light, water and oxygen.

## Graphical abstract

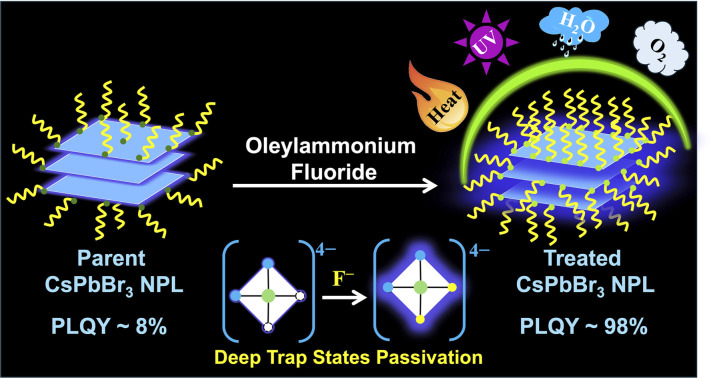

## 1. Introduction

Over the past decade, CsPbX_3_ (where X is Cl, Br or I) perovskite nanocrystals (NCs) have emerged as highly promising materials for future optoelectronic applications. This is owing to their remarkable characteristics, including defect tolerance, clean bandgap properties, high photoluminescence quantum yield (PLQY), tunability of the bandgap controlled by halide ion composition and facile preparation methods.^1,2^ Significant efforts have been devoted to enhancing the PLQY of this material through various innovative strategies such as capping ligand engineering,^3^ B-site doping,^4^ post-synthetic treatments,^5^ and core–shell engineering.^6^ The concerted efforts have led to a significant milestone, with green and red perovskite-based light-emitting diodes (LEDs) achieving an external quantum efficiency (EQE) exceeding 26%.^7,8^ However, the EQE in the blue spectral range (particularly ≤ 460 nm) still lags far behind this notable success.^9^ Urgent and intensified investigation is therefore imperative to surmount this barrier.

In CsPbX_3_ NCs, pure blue PL originates from the compositional engineering of mixed halide NCs,^10,11^ CsPb(Br_*x*_Cl_1−*x*_)_3_, but a fundamental challenge arises from phase instability under photoexcitation and applied electric field conditions, attributed to halide ion migration.^12–15^ Ongoing efforts focus on various passivation strategies to mitigate this issue. Conversely, the quantum confinement of CsPbBr_3_ (CPB) NCs in one dimension yields two-dimensional (2D) nanoplates (NPLs) that emit in the blue region.^16^ Here, the PL peak depends on the thickness (*n*) of the [PbBr_6_]^4−^ layer.^16^ In 2D NPLs, the inorganic slabs of CsPbBr_3_ with a particular thickness are separated by protonated long-chain organic ammonium ions.^16,17^ Thickness-dependent bandgap tunability and large oscillator strength render them promising for optoelectronics.^17^ But these 2D NPLs exhibit less impressive PLQY compared to their 3D analogues as (i) they have a large surface-to-volume ratio, which increases the density of surface traps,^16,18^ (ii) co-ordinated solvent molecules restrict the formation of a defect free crystal structure during crystallization and (iii) during antisolvent-assisted (*i.e.*, acetone and propyl alcohol) synthesis, the loss of capping ligands can generate excess bromide vacancies (V_Br_) on the surface.^18^ Moreover, the phase stability of these 2D structures is compromised, as they are prone to 3D conversion under external perturbations such as light exposure,^19–21^ heat,^22,23^ moisture,^24,25^ polar solvents,^26^ and generally under ambient conditions. Addressing these challenges remains a focus of ongoing research in the field.

Alivisatos and his group introduced the first colloidal synthesis of all inorganic 2D nanoplates (NPLs).^27^ After this seminal contribution, numerous research groups have advanced synthetic methodologies accompanied by comprehensive structural characterization. This progression has led to precise control over shape and thickness,^18,25,28–32^ elucidating charge carrier dynamics,^33–35^ charge carrier extraction,^36–40^ controlled assembly on solid substrates for LED applications,^41–43^ and photodetector applications.^44^ The photoluminescence quantum yield (PLQY) of CPB NPLs is reported to be more than 75% in solution, leading to significant advancements in blue-emitting LED applications.^16^ Both pre- and post-synthetic approaches have been explored to achieve this milestone. The methods include treatments involving long-chain phosphonate ligands,^45^ short-chain sulfonates,^44,46^ acids and amines,^47,48^ amine-free synthesis,^49^ as well as the utilization of cross-linking,^50^ semiconducting,^51^ and multidentate ligands,^52^ core–shell treatment,^53^ doping^54,55^ along with various inorganic^18,56–58^ and polymeric zwitterionic bromide salts^59^ or direct HBr^60^ treatment. These treatments are effective as they (i) establish stable coordination bonds between unsaturated surface Pb atoms and heteroatoms present in the capping ligands and/or, (ii) eliminate the Br^−^ vacancy-related trap states, which not only enhance PLQY but also augment the NPLs' resilience against external factors such as UV light exposure, air and water. However, further attention and progress are still needed as blue perovskite-based LEDs are still far behind the success achieved by green and red LEDs. Also, the reported strategies often prove costly and time-consuming and, they fail to address all challenges regarding external factors like UV exposure, heat, air and water simultaneously.

To simultaneously enhance the PLQY and stability of CPB NPLs, we hypothesized that incorporating highly electronegative fluorine (F) atoms on the NPL's surface could effectively address these challenging issues as F^−^ ions may repair the bromide vacancy and form a Pb–F terminated surface. The Pb–F bond energy is the highest among all Pb–X bonds which will be highly beneficial for an ultrastable NPL surface. Recently, researchers have also introduced different fluoride salts or directly used hydrofluoric acid to reduce thermal-assisted PL quenching and to boost the extrinsic stability of 3D perovskite NCs.^61–65^ Building on this hypothesis, we successfully synthesized oleylammonium fluoride (OAmF) salt for the first time as per the literature by reacting oleylamine with hydrofluoric acid in an equimolar ratio. Subsequently, we treated the CPB NPLs (thicknesses of *n* = 3 and *n* = 4) with this OAmF salt. The NPLs exhibited several significant improvements after this post-treatment, *i.e.*, (i) the incorporation of F^−^ ions on the surface effectively passivated detrimental Br^−^ vacancy-related deep trap states, boosting the PLQY by more than 11 times, reaching near unity and (ii) the NPL's facets were stabilized through strong electrostatic interactions and hydrogen bonding due to the enhanced interaction between primary oleylammonium cations and halide ions (Br^−^ and F^−^). This enhanced stabilization prevented the undesirable 2D to 3D phase transformation of the NPLs when exposed to external factors such as heat, UV irradiation, water, ambient conditions and solvent polarity. Strengthening the Pb–F bond over the Pb–Br bond probably created a more resilient and stable F-rich surface, potentially mitigating the drawbacks associated with CPB NPLs.

Compared to previous F^−^ post-synthetic treatments, our work has two key advantages: (i) unlike previous studies^61–65^ where the anionic fluoride (F^−^) is typically bound to a metal or quaternary ammonium cation, in the present work it is bound to a primary ammonium cation having an –NH_3_^+^ head group (*i.e.*, oleylammonium cation) and (ii) while prior treatments were focused only on 3D CsPbBr_3_ NCs, our post-synthetic treatment is specifically tailored for its 2D analogues (*i.e.*, blue-emitting CsPbBr_3_ NPLs), which suffer from numerous challenges like low quantum yield, phase instability and structural vulnerability under external perturbations. Here, the F^−^ and –NH_3_^+^ head groups have critical roles in enhancing the optical properties and stability of the NPLs, respectively.

## 2. Results and discussion

### 2.1. Post-synthetic treatment of CsPbBr_3_ NPLs with OAmF

**Scheme 1:**
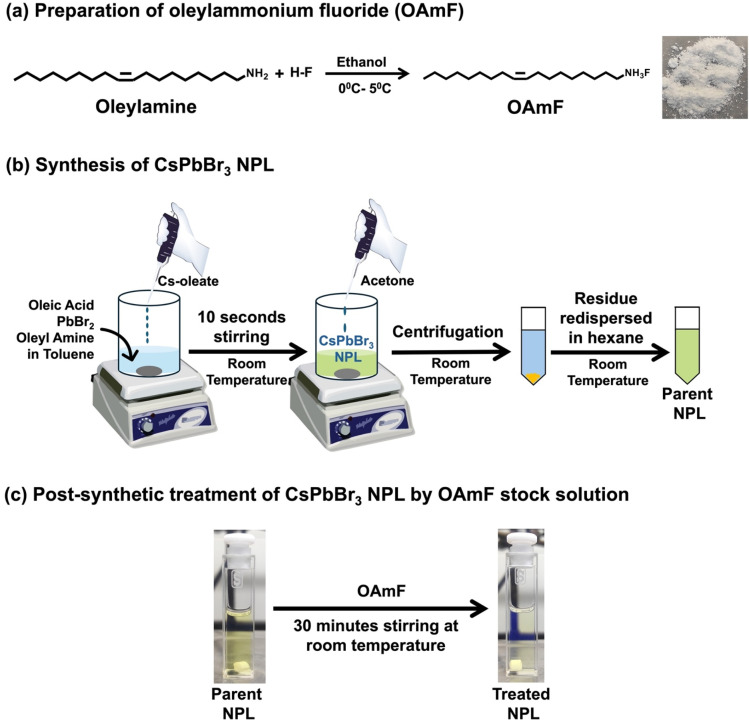
Schematic illustration for the synthesis of oleylammonium fluoride (OAmF) treated CsPbBr_3_ NPLs. (a) Synthesis scheme of oleylammonium fluoride (OAmF) salt. The photographic image shows the synthesized OAmF salt after preparation. (b) Stepwise synthesis of blue-emitting CsPbBr_3_ NPLs (parent) at room temperature. (c) Post-synthetic treatment of the parent NPL with OAmF stock solution at room temperature under open atmospheric conditions. The photographic images show the parent NPL and treated NPL, highlighting the colour change of the parent NPL from pale yellow to pale blue after treatment.

To achieve the blue PL spectral region, we targeted to synthesize quantum confined CPB NPLs with thicknesses of *n* = 3 and *n* = 4 following a previous report.^18^ The detailed synthesis procedure is provided in the Experimental section, and the synthesized NPL is abbreviated as the “parent NPL” throughout this manuscript (see Scheme 1 and the Experimental section for details). First, we prepared oleylammonium fluoride (OAmF) salt by reacting oleylamine and hydrofluoric acid in an equimolar ratio (see Scheme 1a and the Experimental section for details). In brief, our synthesis method involves the addition of the pre-synthesized Cs-oleate precursor at room temperature to a vigorously stirred toluene solution containing lead bromide, oleic acid, and oleyl amine. We added acetone to initiate the formation of NPLs, followed by centrifugation, and the residual part was dispersed in hexane as the parent NPL (see Scheme 1b and the Experimental section for details). Then we post-synthetically treated the parent NPL with a specific volume of OAmF stock solution in hexane at room temperature (see Scheme 1c and the Experimental section). After treatment, the parent NPL changes its colour from pale yellow to pale blue (see Scheme 1c), and this is designated as the “treated NPL” in this manuscript. The stepwise synthetic pathway for the treated NPL by OAmF post-synthetic treatment is summarized in Scheme 1.

### 2.2. Morphology and crystal structure of the parent and treated NPL with surface functionalization with OAmF

#### 2.2.1. PXRD analysis

**Fig. 1:**
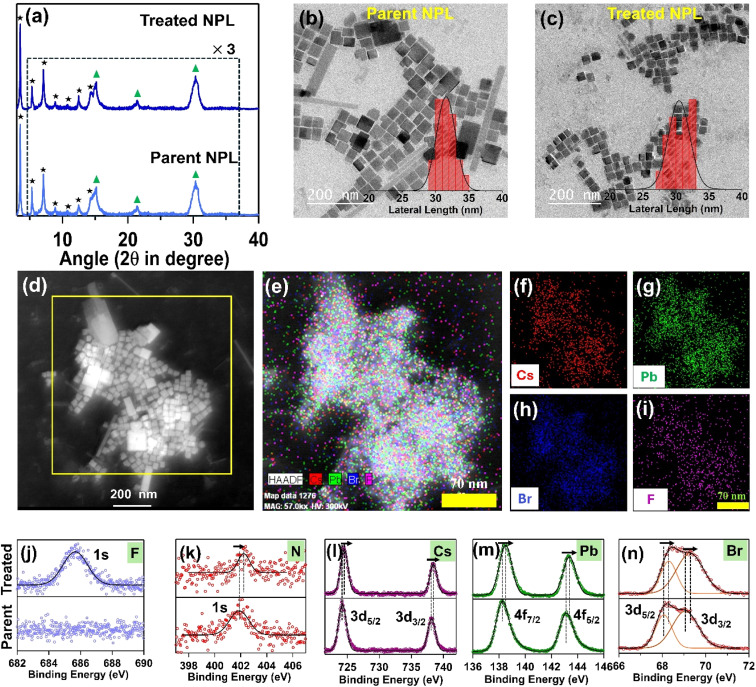
(a) PXRD pattern of the parent and treated CsPbBr_3_ NPL (*n* = 3). The equidistant 2*θ* reflection indicated by an asterisk symbol for both NPLs represents the stacking of the RP NPL structure. The 2*θ* reflections indicated by a triangle symbol for both NPLs originate from the CsPbBr_3_ unit cell. The TEM images of the (b) parent and (c) treated CsPbBr_3_ NPL (*n* = 3). (d) High angle annular dark field (HAADF) image of the treated CsPbBr_3_ NPL (*n* = 3). (e) The overall elemental mapping from the selected portion of (d) showing the presence of Cs, Pb, Br, and F elements throughout the NPL. The individual elemental mapping of (f) Cs, (g) Pb, (h) Br and (i) F elements. The high-resolution XPS of the parent (lower panel) and treated (upper panel) CsPbBr_3_ NPL (*n* = 3) in (j) F-1s, (k) N-1s, (l) Cs-3d, (m) Pb-4f and (n) Br-3d regions.

We performed powder X-ray diffraction (PXRD), transmission electron microscopy (TEM) and X-ray photoelectron spectroscopy (XPS) measurements to characterize the parent and treated NPL. The PXRD measurement for the *n* = 3 parent NPL showed distinct lower angle reflection peaks at 3.5°, 5.3°, 7.0°, 8.8°, 10.6°, 12.4° and 14.2° with three characteristic peaks at 15.0°, 21.4° and 30.1° corresponding to the (100), (110) and (200) crystallographic planes of the cubic CsPbBr_3_ crystal (see Fig. 1a). The equidistant lower angle reflections (Δ2*θ* = 1.8°) indicate that the NPLs are stacked and the average spacing between two NPL units is estimated to be ∼4.8 nm which is the sum of 1.9 nm for three [PbBr_6_]^4−^ octahedral layers (as the height of the one [PbBr_6_]^4−^ octahedron is ∼0.66 nm) and 2.9 nm for the oleylammonium ligand, with some interdigitation as observed in the previous report.^52^ Therefore, the PXRD pattern confirms the generation of phase pure (*n* = 3) Ruddlesden Popper (RP) stacked NPL structures in colloidal solution. After the post-synthetic treatment, the PXRD pattern of the *n* = 3 treated NPL was found to be unaffected with no new peaks observed, indicating that the treatment did not alter the underlying crystal structure (see Fig. 1a). Furthermore, we conducted small-angle X-ray scattering (SAXS) measurements to identify the low-angle diffraction peaks that was not detectable using PXRD due to its limited resolution. For both samples, the first peak appeared at 1.8°, followed by a second peak at 3.5° (see Fig. S1 of the ESI†). These peaks align perfectly with the PXRD diffraction pattern, showing equidistant reflections at 1.8°, confirming that the inorganic layer's thickness is approximately 1.9 nm corresponding to *n* = 3. Finally, we have provided a schematic representation of the stacked RP NPL structure in Fig. S1b of the ESI,† illustrating the relevant thickness of the inorganic lattice and spacer ligands for better clarity and understanding.

#### 2.2.2. HRTEM analysis

From the TEM image, we observed mainly square-shaped structures for the *n* = 3 parent NPL with an average lateral length of 31.5 ± 3 nm (see Fig. 1b). In our case, the obtained NPLs were stacked in a face-down arrangement instead of an edge-to-edge stack, and we believe that the fast evaporation of our dispersion solvent (*i.e.*, hexane) controlled this arrangement as mentioned by Bawendi and co-workers.^41^ From the high-resolution (HR) TEM image, we isolated (100) and (110) crystallographic planes having interplanar distances of ∼0.58 nm and ∼0.41 nm, respectively, consistent with the previous report for the cubic phase (see Fig. S2a and b in the ESI†).^66,67^ After OAmF treatment, we also observed a similar morphology of the NPL with a lateral length distribution of 30.5 ± 2.5 nm (see Fig. 1c). The treated NPL also shows similar crystallographic planes like the parent NPL (see Fig. S3a–d in the ESI†). The FFT pattern obtained from the HRTEM images and high-angle annular dark field (HAADF) imaging confirms the high crystallinity of the NPLs (see Fig. 1d). The elemental mapping of a selected portion of the HADDF image confirms the incorporation of F^−^ ions into the NPL with compositional Cs, Pb, and Br elements (see Fig. 1e–i).

#### 2.2.3. XPS analysis

We performed XPS to study the changes in the surface environments of the *n* = 3 parent and treated NPL. Due to F^−^ ion incorporation, the treated NPL showed a characteristic F-1s peak at 685.6 eV (see Fig. 1j).^61^ The direct incorporation of the most electronegative F element affects the chemical environment of every constituent element, causing the binding energy to shift towards a higher value for the treated NPL compared to the parent NPL (see Fig. 1j and Table S1 of the ESI†). The shift of the N-1s binding energy (for –NH_3_^+^) toward a higher value for the treated NPL corresponds to stronger H-bonding interaction between protons of ammonium head and surface F^−^ and Br^−^ ions (see Fig. 1k and Table S1 of the ESI†).^47,68^ We obtained two distinguished peaks for Cs^+^ ions at 724.2 eV (3d_5/2_) and 738.1 eV (3d_3/2_) for the parent *n* = 3 NPL, which shifted towards a higher binding energy of 0.3 eV for the treated *n* = 3 NPL (see Fig. 1l and Table S1 of the ESI†).^61^ This observation underscores a change in the chemical environment of the Cs atom in the *n* = 3 treated NPL in comparison to the parent NPL. The repair of Br^−^ vacancies by F^−^ on the [CsBr]-terminated facet is likely responsible for the alteration in the chemical environment of Cs, as suggested in the previous report.^69^ Similarly, we observed a shift in binding energy of Pb^2+^ towards a higher value (by 0.3 eV) in the treated NPL in comparison to the parent one. This also indicates the formation of new Pb–F bonds (see Fig. 1m and Table S1 of the ESI†).^70^ Additionally, we observed a similar shift of 0.23 eV towards higher binding energy for the Br-3d peaks, attributed to the significant change in the chemical environment following the insertion of F^−^ on the surface (see Fig. 1n and Table S1 in the ESI†). We also determined the Pb : Br elemental ratio for the *n* = 3 parent NPL to be 1.00 : 2.50, which suggests that the parent NPL suffers from predominant halide vacancy-related defects. However, for the treated NPL, the Pb : (Br + F) ratio becomes 1.00 : 3.12, corresponding to a halide-rich surface arrangement where the Br^−^ vacancy is repaired by the F^−^ ion. For the *n* = 4 NPL, before and after treatment, similar observations were made (please see ESI Note S1, Fig. S4, S5 and Table S2 in the ESI†)

### 2.3. Change in optical properties of the parent and treated NPL

#### 2.3.1. Steady state measurements

**Fig. 2:**
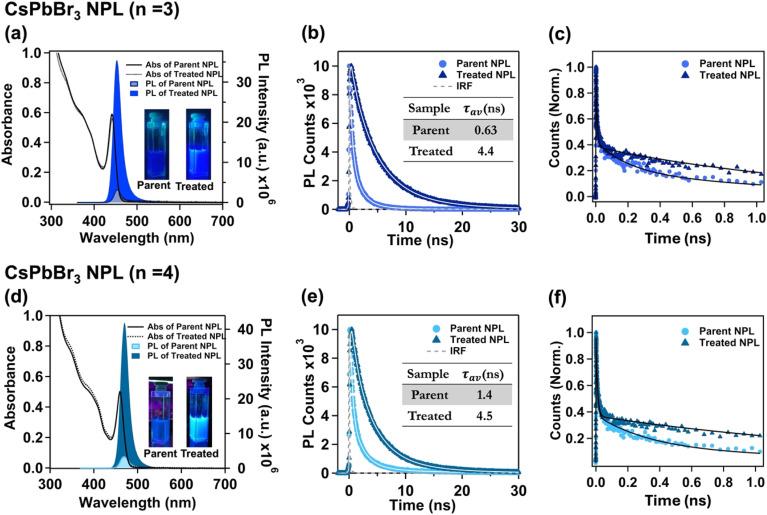
Optical properties of CsPbBr_3_ NPLs (*n* = 3) before and after OAmF treatment. (a) The UV-visible absorption and PL spectra. (b) PL decay. (c) Femtosecond time-resolved PL decay. (d) The UV-visible absorption and PL spectra, (e) PL decay and (f) femtosecond time-resolved PL decay of parent and treated CsPbBr_3_ NPLs (*n* = 4). All PL spectra were recorded upon exciting the sample at 350 nm. The nanosecond and femtoseconds PL transients were recorded by exciting the sample at 375 nm and 400 nm, respectively.

We investigated the change in the optical properties of the NPLs before and after the treatment. Remarkably, the absorption and photoluminescence (PL) peak properties of both *n* = 3 and *n* = 4 NPLs remained unaltered before and after treatment, up to a specific volume of OAmF stock solution (see Fig. 2a, d and Table 1). Notably, this post-treatment effect was observed to be specific to a particular volume of OAmF stock solution for the best PLQY result (20 μL for the *n* = 3 parent NPL and 15 μL for the *n* = 3 parent NPL). The distinct excitonic absorption peak, around 441.4 nm for the *n* = 3 NPL signifies the quantum confinement within the NPLs. The PL peak position at 453 nm indicates the formation of a specific *n* = 3 NPL (see Fig. 2a and Table 1). These NPLs showed a PLQY of 8 ± 2%. Following treatment with 20 μL of OAmF stock solution, a remarkable surge in PL intensity (approximately 11-fold) was observed without any discernible impact on the excitonic peak, PL peak and full width at half maximum (FWHM) (∼21 nm) (see Fig. 2a and Table 1). The PLQY of the *n* = 3 treated NPL was measured to be 85 ± 2%. Conversely, the parent *n* = 4 NPLs displayed a sharp excitonic absorption peak and a PL peak at ∼459.8 nm and 470 nm, respectively, with a PLQY of 9 ± 2% (see Fig. 2d and Table 1). After treatment with 15 μL of OAmF stock solution, the PL intensity surged approximately 12.7 times achieving a near unity PLQY (98 ± 2%), that too without any apparent alteration in the absorption peak, PL peak, and FWHM (22 nm) (see Fig. 2d and Table 1). A striking difference in PL under 365 nm UV excitation was observed for both the parent and treated NPL (*n* = 3 and *n* = 4), confirming the effective surface passivation by small F^−^ ions to repair the Br^−^ related vacancy trap states (see inset images in Fig. 2a and d). Upon incremental addition of OAmF stock solution, the PL intensity for both *n* = 3 and *n* = 4 treated NPLs increased with no red shift in the excitonic and PL peaks, discarding the possibility of 3D-NC formation. This observation underscores that the effective F^−^ surface passivation does not induce alterations in the bandgap of the parent NPL. However, after reaching a certain volume threshold of OAmF stock solution, the excitonic absorption and PL peaks undergo a red shift, accompanied by a decrease in PL intensity (see Fig. S6a, b, d, S7a, b, d and Tables S3, S4 in the ESI†). This characteristic red shift, coupled with the reduction of PL likely signifies that the excessive F^−^ treatment disrupts the octahedral stability of the [PbX_6_]^4−^ structure (guided by the octahedral *μ* factor), thereby promoting particle coalescence and/or structural degradation.^62^

**Table 1:** Optical properties of the parent and treated CsPbBr_3_ NPL with thicknesses of *n* = 3 and *n* = 4^a^

Sample	Excitonic peak (nm)	PL max (nm)	PLQY	*τ*_avg_ (ns)	*k*_r_ (ns^−1^)	*k*_nr_ (ns^−1^)
Parent NPL (*n* = 3)	441.4	453	8 ± 2%	0.63	0.126	1.46
Treated NPL (*n* = 3)	441.4	453	85 ± 2%	4.4	0.193	0.034
Parent NPL (*n* = 4)	459.8	470	9 ± 2%	1.4	0.06	0.65
Treated NPL (*n* = 4)	459.8	470	98 ± 2%	4.5	0.22	0.004

a *τ*_avg_ is the average lifetime, *k*_r_ is the radiative rate constant, and *k*_nr_ is the non-radiative rate constant.

#### 2.3.2. Time-resolved measurements for charge carrier dynamics

From the time-correlated single photon counting (TCSPC) measurement we observed that both *n* = 3 and *n* = 4 parent NPLs follow complex tri-exponential decay kinetics (see Fig. 2b, e and Tables S5, S6 of the ESI†). For the *n* = 3 parent NPL, the average lifetime (*τ*_avg_) was measured to be 0.63 ns, where the lifetime components with their amplitudes are 210 ps (*τ*_1_, 66.7%), 1.1 ns (*τ*_2_, 27.4%) and 3.3 ns (*τ*_3_, 5.9%) (see Table S5 of the ESI†). On the other hand, the average lifetime of the *n* = 4 parent NPL was measured to be 1.4 ns with lifetime components of 340 ps (*τ*_1_, 50%), 1.8 ns (*τ*_2_, 37.5%) and 4.7 ns (*τ*_3_, 12.5%) (see Table S6 of the ESI†). We assign *τ*_1_ to the non-radiative recombination of charge carriers due to the presence of deep trap states, *τ*_2_ to band-to-band excitonic recombination and *τ*_3_ to surface trap assisted excitonic recombination as reported in previous studies.^25,34,71–73^ Upon treatment with the OAmF stock solution, the amplitude of *τ*_1_ decreased with an concomitant increase in the *τ*_2_ and *τ*_3_ amplitudes, resulting in an increase in the *τ*_avg_ for both the cases (see Fig. S6c, d, S7c, d and Tables S5, S6 in the ESI†). Upon treatment with the optimal volume of OAmF stock solution (20 μL for *n* = 3 and 15 μL for *n* = 4 as already mentioned), the PL transient exhibited bi-exponential decay kinetics with an average lifetime of 4.4 ns (*τ*_2_: 2.2 ns (33.3%) and *τ*_3_: 5.5 ns (66.7%)) and 4.5 ns (*τ*_2_: 2.2 ns (34.5%) and *τ*_3_: 5.7 ns (65.5%)) for the *n* = 3 treated NPL and *n* = 4 treated NPL, respectively (see Fig. 2b–e and Tables S5, S6 of the ESI†). The post-treatment reduction in the non-radiative rate constant (42.9 times for the *n* = 3 NPL and 162.5 times for the *n* = 4 NPL) indicates a significant reduction in trap state density (especially deep traps)^74^ (see Table 1 and ESI Note S2 of the ESI†).

To further explore the existence of deep trap states in the parent NPL and their subsequent elimination through treatment, we measured fluorescence transients at early times using the femtosecond fluorescence up-conversion technique having sub-ps time resolution. As charge carrier trapping occurs on the ps timescale, the femtosecond time-resolved experiment provides a profound understanding of the ultrafast charge carrier trapping. The PL transients obtained from the experiments were best fitted with three lifetime components for both *n* = 3 and *n* = 4 parent NPLs. The lifetime components and their amplitudes are 6.0 ps (61.8%), 200 ps (20%) and >1 ns (18.2%) for the *n* = 3 parent NPL, whereas they are 8.5 ps (66%), 350 ps (25%) and >1 ns (9%) for the *n* = 4 parent NPL (see Fig. 2c and f). Among these lifetime components, the fastest lifetime component (<10 ps) with maximum contribution is attributed to charge carrier trapping from the band edge to dark shallow trap states; these shallow trap states are assumed to be located close to the band edge within a few meV.^75^ The lifetime components of 200 ps and 350 ps for *n* = 3 and *n* = 4 treated NPLs, respectively, possibly originate from carrier trapping in deep trap states matching with the TCSPC fittings, and the >1 ns component for both cases is attributed to band-to-band excitonic recombination.^75^ Interestingly, we observed that the lifetime component related with the deep trap states was completely eliminated for the treated NPL and the measured PL transients could be best fitted with a bi-exponential function. For *n* = 3 treated NPLs, the lifetime components are found to be 7.1 ps (65.5%) and 1.5 ns (34.5%), whereas for *n* = 4 treated NPLs, the lifetime components are measured to be 9.5 ps (65%) and 1.9 ns (35%) (see Fig. 2c and f).

#### 2.3.3. Possible mechanism for the repair of deep trap states

**Scheme 2:**
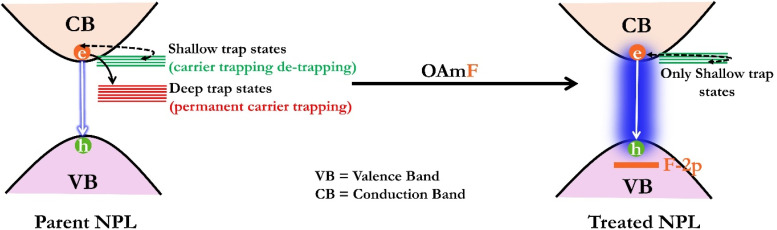
Schematic representation of oleylammonium fluoride post-synthetic treatment on CsPbBr_3_ NPLs to reveal the enhancement in PLQY by passivation of deep trap states.

The efficacy of F^−^ induced surface passivation through OAmF treatment is confirmed through XPS and elemental analysis *via* HRTEM. This process involves the repair of Br^−^ vacancies by smaller F^−^ ions, which not only diminishes the most detrimental halide vacancy-related trap sites within NPLs, but also establishes a stable fluoride-passivated surface. Moreover, the TRPL measurements confirm the elimination of deep trap states on OAmF treatment. Although this passivation eliminates deep trap states within the NPLs, the treated NPLs still have shallow trap states that likely reside near the conduction band. Ideally, quantum-confined 2D nanoplatelets (NPLs) should exhibit mono-exponential PL transients, as they show exciton-dominated photophysics. However, in our case, even after achieving near-unity PLQY for *n* = 4 treated NPLs, the bi-exponential PL transient was still observed, which confirms the presence of shallow trap states, as seen in previous reports.^74,76^ These shallow trap states prolong the lifetime due to charge carrier trapping and de-trapping.^76^ With a schematic representation, we have summarized this fact in Scheme 2.

### 2.4. Stability against external environmental factors

In previous reports, we observed that 2D perovskite NPLs are prone to transformation into 3D NCs under external perturbations such as heat, UV irradiation, moisture, atmospheric conditions or changes in the polarity of the dispersion solvent.^19–21,23,26^ This transformation is triggered by the easy detachment of capping ligands from the surface, leading to the coalescence of the NPLs and a consequent loss of their quantum confinement properties. Such phase transformations compromise the crystalline phase of the NPLs, making them less suitable for device-related applications. We have evaluated the effectiveness of our post-synthetically treated NPL against various environmental factors, including heat, UV irradiation, water, ambient conditions and solvent polarity of the medium, which are presented below.

#### 2.4.1. Stability against heat

**Fig. 3:**
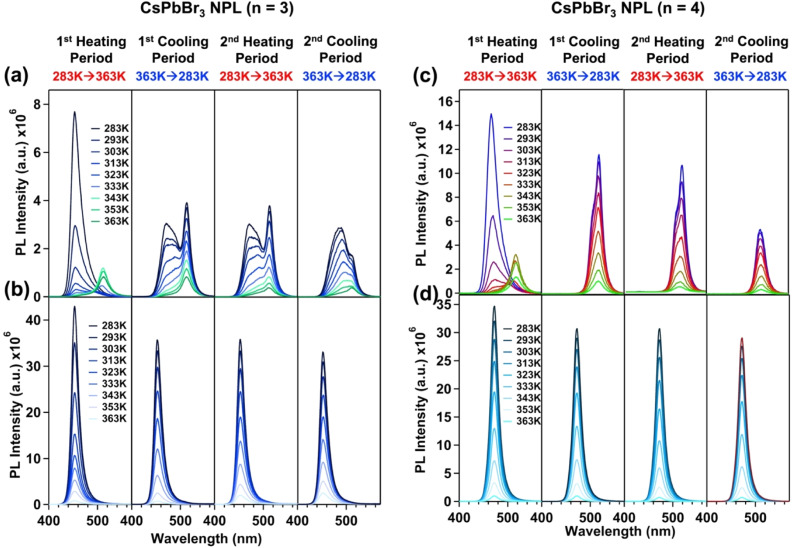
The PL stability of parent and treated CsPbBr_3_ NPLs against heat (283 K to 363 K) in solution. The PL spectra of (a) parent CsPbBr_3_ NPLs (*n* = 3) (left upper panel), (b) treated CsPbBr_3_ NPLs (*n* = 3) (left lower panel), (c) parent CsPbBr_3_ NPLs (*n* = 4) (right upper panel), and (d) treated CsPbBr_3_ NPLs (*n* = 4) (right lower panel) with an increase in the temperature (1st heating period), decrease in the temperature (1st cooling period), increase in the temperature (2nd heating period), and decrease in the temperature (2nd cooling period). All PL spectra were recorded by exciting the sample at 350 nm.

We investigated the heat tolerance properties of both the parent and treated NPLs by measuring the temperature dependent PL in toluene. For this, we recorded two cycles of temperature-dependent PL, with each cycle consisting of one heating period in the forward direction (283 K to 373 K) and one cooling period in the backward direction (373 K to 283 K). In the first heating period, the *n* = 3 parent NPL was totally transformed into 3D NCs where the PL peak near 453 nm disappeared with the formation of a new peak at 511 nm (see Fig. 3a), which is similar to the previous observation.^22^ After the first cooling period, we observed two PL maxima near 511 nm and 471 nm at 283 K (see Fig. 3a). This observation indicates that only a fraction of the 3D NCs is converted back into the 2D phase with a thickness of *n* = 4. After the second heating period, we observed predominantly 3D NCs at 363 K. Again, after the second cooling period, we observed the generation of a new PL peak at 490 nm with a hump around 511 nm, which refers to the existence of *n* = 5 NPLs and 3D NCs at 283 K. For *n* = 4 parent NPLs, we observed a total irreversible phase transformation of 2D NPLs into 3D NCs after two cycles (see Fig. 3c). Thus, the parent NPLs underwent an irreversible 2D to 3D phase transition when subjected to heat, resulting in the loss of their quantum confinement. In contrast, the treated NPLs (both *n* = 3 and *n* = 4) exhibited only PL quenching with increasing temperature, without any shift in the PL maximum (453 nm and 470 nm for *n* = 3 and *n* = 4 treated NPLs, respectively) and, importantly, without undergoing the undesired 2D to 3D phase transition (see Fig. 3b and d). This indicates that the treated NPLs retained their quantum confinement and structural integrity under thermal stress, demonstrating the effectiveness of our modification. The decrease in PL with increasing temperature is attributed to the thermal quenching of PL based on earlier reports.^61,77^ For *n* = 3 treated NPLs, the PL recovery after the first and second cooling period was 83% and 77%, respectively. In contrast, for *n* = 4 treated NPLs, the recoveries were 88% and 83%, respectively (see Fig. S8a and b of the ESI†).

**Fig. 4:**
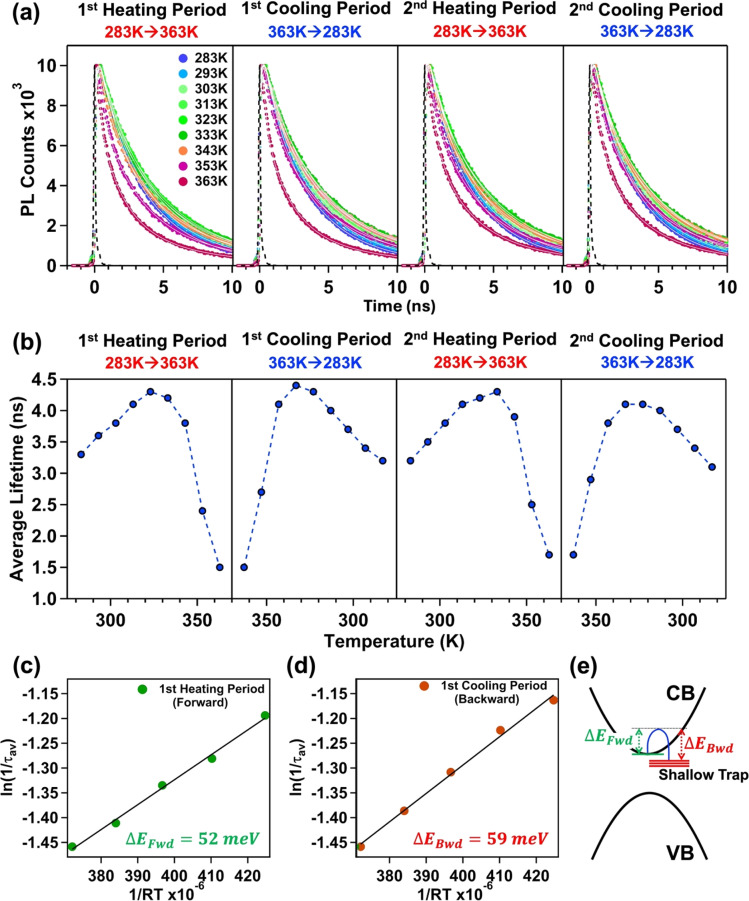
The change in (a) PL transients and (b) corresponding average lifetime (ns) of the treated CsPbBr_3_ NPL (*n* = 3) with an increase in temperature (1st heating period), a decrease in temperature (1st cooling period), an increase in temperature (2nd heating period), and a decrease in temperature (2nd cooling period). Determination of the activation energy of carrier trapping and de-trapping (Δ*E*_trap_) from the Arrhenius equation (eqn (1)) in the temperature range of 283 K to 323 K for the (c) 1st heating period and (d) 1st cooling period. (e) Schematic representation of the location of shallow trap states from the conduction band minima.

To delve deeper into the charge carrier dynamics of the treated NPL during heating and cooling cycles, we conducted temperature-dependent TRPL measurements for *n* = 3 treated NPLs. With an increase in the temperature up to 333 K (1st and 2nd heating periods), we observed a gradual increase in the average lifetime (see Fig. 4a and b). Concomitantly, the amplitude of the excitonic lifetime component (*τ*_2_) decreased, while the surface trap assisted lifetime component (*τ*_3_) increased (see Tables S7 and S9 of the ESI†). This observation suggests that the involvement of shallow trap states near the excitonic states prolongs the average lifetime *via* the carrier trapping/de-trapping process as this is a thermally activated process. This observation is consistent with previous reports by Samanta and co-workers.^74,76^ However, beyond 333 K, a further increase in the temperature led to a reduction in the average lifetime (see Fig. 4a and b). At 353 K and 363 K, we observed a complex triexponential PL decay with a shorter lifetime component of ∼200–300 ps having a large amplitude (see Tables S7 and S9 of the ESI†). The result indicates the involvement of deep trap states in addition to the existing shallow surface trap states. This suggests that the surface of the treated NPL undergoes significant changes beyond 333 K, probably due to the substantial removal of surface oleylammonium cations, halide ion migration (specifically Br^−^), crystal deformation, *etc.*, which introduce deep trap states with structural defects into the crystal. Therefore, we conclude that charge carrier recombination in the treated NPLs occurs primarily *via* shallow trap states up to 333 K. Beyond this temperature, both shallow and newly generated deep trap states with structural defects predominate in the carrier recombination process.

Conversely, decreasing the temperature completely reversed this trend. With a decrease in the temperature, an increase in the average lifetime up to 343 K was observed, followed by a gradual decrease in the average lifetime till 283 K (see Fig. 4a, b and Tables S8, S10 of the ESI†). The average lifetime follows a complex triexponential decay up to 353 K, and beyond that, it follows bi-exponential decay kinetics with an increasing contribution from the excitonic lifetime component and a decreasing contribution from the surface trap assisted lifetime component (see Tables S8 and S10 of the ESI†). This reversibility suggests that after reaching the threshold temperature of 343 K, the deep trap states were eliminated *via* the resorption of oleylammonium cations and halide anions into the NPL's surface (we observed the recovery of PL during the cooling period); the excitonic recombination becomes predominant in the charge carrier trapping process due to the decrease in temperature.

From the average lifetime values during the first heating (*i.e.*, from 283 K to 323 K) and cooling (from 323 K to 283 K) periods, we determined the activation energy for charge carrier trapping (Δ*E*_Fwd_) into shallow trap states and de-trapping (Δ*E*_Bwd_) from the shallow trap states, respectively, by using the simple Arrhenius equation as follows1
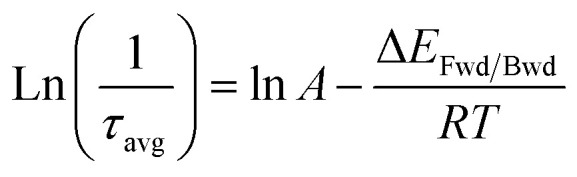


According to eqn (1), we plotted 
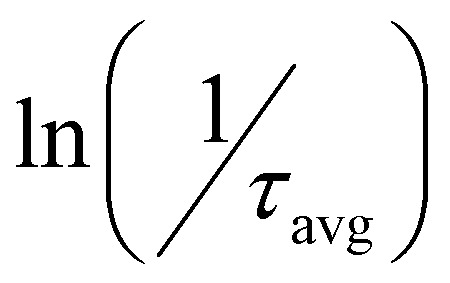
*vs.*
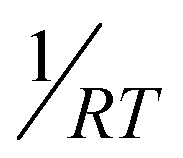
 and the activation energy for carrier trapping and de-trapping was measured to be 52 meV and 59 meV, respectively (please see Fig. 4c and d). We have schematically demonstrated the forward activation energy (Δ*E*_Fwd_), backward activation energy (Δ*E*_Bwd_) and the presence of shallow trap states near the conduction band edge in Fig. 4e. From this, we can clearly determine that the shallow trap states are located 7 meV below the conduction band edge. While our TRPL measurements confirm the existence of shallow trap states in the treated NPL, the temperature-dependent TCSPC measurements provide an insight into the energetic position of these states relative to the conduction band edge. Socie *et al.* first provided spectroscopic evidence of shallow trap states in 2D CsPbBr_3_ NPLs.^75^ Our report further extends their concept and for the first time provides a better understanding of the closely lying shallow trap states near the band edge with a reliable magnitude of energy.

We evaluated the heat tolerance of both the parent and treated *n* = 3 NPL in film form, considering their potential application in LED fabrication. The films were heated on a hot plate at a constant temperature of 343 K for 45 minutes. We continuously monitored the PL of the films by capturing images under 365 nm UV excitation. For the parent NPL, a green PL appeared after 30 minutes of heating, indicating a phase transition from the 2D NPL to the 3D NC (see Fig. S9a of the ESI†). In contrast, the treated NPL exhibited blue PL for up to 45 minutes confirming the phase stability of the treated NPL in film form (see Fig. S9b of the ESI†).

#### 2.4.2. Stability against UV light exposure

Photodegradation and photo-induced phase transformation of the 2D perovskite NPL crystal structure under continuous UV irradiation is a crucial problem, well-documented in the literature.^19–21^ The crystal structure of colloidal CPB NPLs also degraded under high energy UV exposure, losing its quantum confinement due to the detachment of weakly bound acid-amine ligands, finally transforming into 3D NCs.^16^

**Fig. 5:**
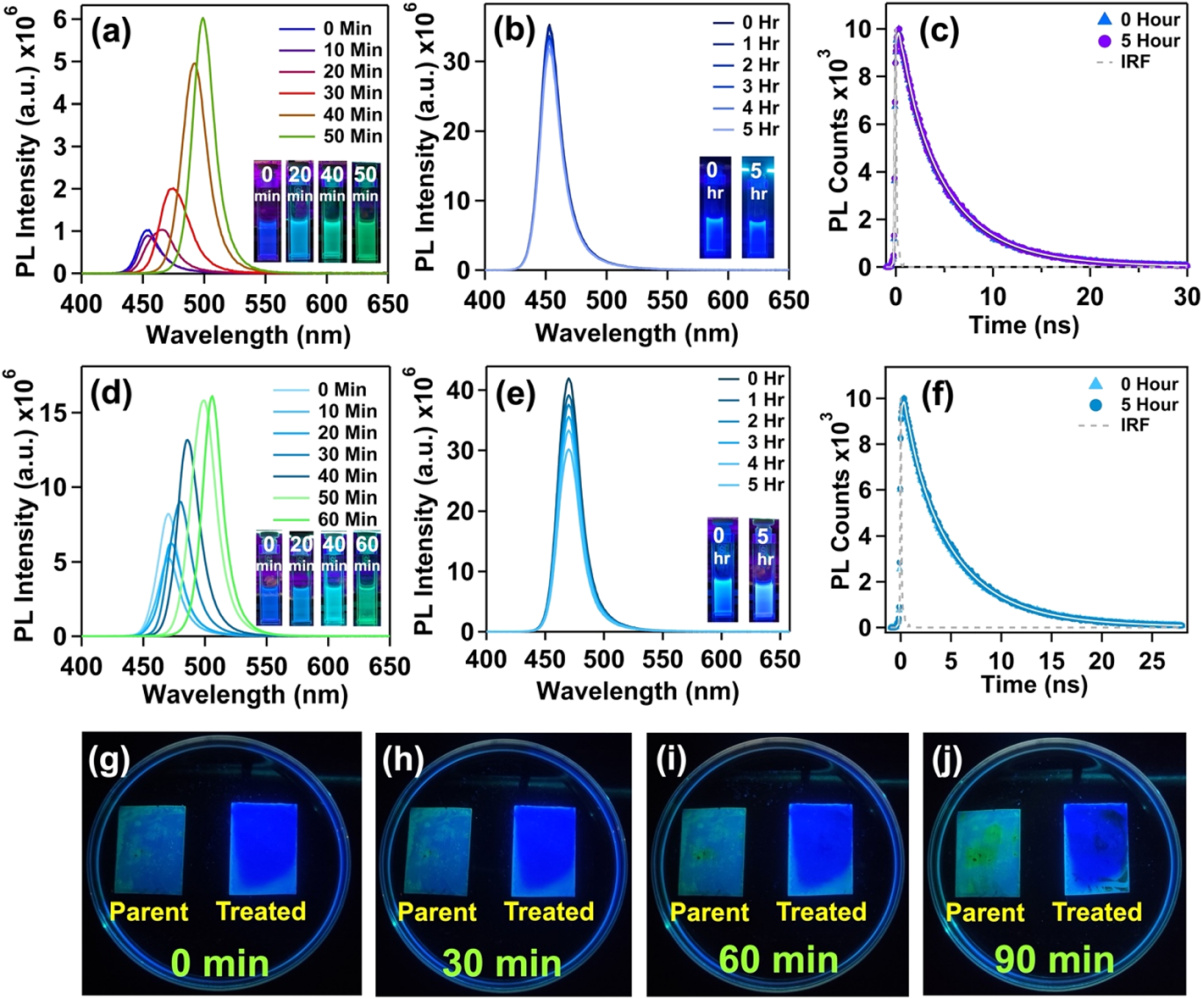
The comparison of UV stability and water stability of parent and treated NPLs. The change in PL of (a) parent NPLs (*n* = 3) and (b) treated NPLs (*n* = 3) and (c) PL transients of treated NPLs (*n* = 3) with continuous 365 nm UV excitation under open atmospheric conditions. The inset photographic images in (a) and (b) represent the change in the PL colour of the parent NPL (*n* = 3) and treated NPL (*n* = 3), respectively, under 365 nm UV excitation. The change in PL of (d) parent NPLs (*n* = 4) and (e) treated NPLs (*n* = 4) and (f) PL transients of treated NPLs (*n* = 4) with continuous 365 nm UV excitation under open atmospheric conditions. The inset photographic images in (d) and (e) represent the change in the PL colour of the parent NPL (*n* = 4) and treated NPL (*n* = 4), respectively, under 365 nm UV excitation. (g)–(j) The drop-cast films of parent (left) and treated (right) CsPbBr_3_ NPLs (*n* = 3) were immersed in 5 mL of deionized water in a Petri dish to check the water stability, where the PL of the film was monitored by 365 nm UV excitation.

In the present case also, the parent blue-emitting *n* = 3 and cyan-emitting *n* = 4 NPLs gradually transformed into green-emitting 3D CsPbBr_3_ NCs under continuous 365 nm UV exposure. For *n* = 3 parent NPLs, the PL peak around 454 nm gradually shifted to 500 nm after 50 minutes of continuous UV exposure, accompanied by a red shift (see Fig. 5a). But, for *n* = 3 treated NPLs, we did not observe any red shift of the PL peak at 453 nm even after 5 hours of continuous UV exposure (see Fig. 5b). This suggests the prevention of the undesirable 2D to 3D phase transformation in the treated NPL. Also, we observed only an 8% decrease in initial PL after 5 hours of continuous UV exposure for *n* = 3 treated NPLs indicating its exceptional photostability. After 5 hours of continuous exposure, the similar average lifetime with unaffected excitonic recombination and shallow trap assisted lifetime components suggests that no new defect states were generated (see Fig. 5c and Table S11 of the ESI†). Similarly, for *n* = 4 parent NPLs, we observed a red shift from 470 nm to 506 nm after 60 minutes of continuous UV exposure, whereas the *n* = 4 treated NPLs retain 86% PL after 5 hours of UV exposure without affecting the PL maxima at 470 nm (see Fig. 5d and e). The unaffected average lifetime in time resolved measurements for *n* = 4 treated NPLs followed a similar trend to that of *n* = 3 treated NPLs (see Fig. 5f and Table S11 of the ESI†). Overall, the OAmF treated NPL restricts the detachment of strongly bound surface capping ligands and halide ion migration, thus inducing photostability, which is highly desirable for optoelectronic applications.

#### 2.4.3. Stability against water

We also checked the water stability of the parent and treated *n* = 3 NPL by immersing the film in water and capturing images at different times to probe the change in PL under 365 nm UV excitation. After immersing the parent NPL film in water, we immediately observed a faint sky-blue PL from the film, which slowly changed into green and sky-blue PL (see Fig. 5g–j), probably due to the formation of thicker 2D NPLs and a phase transition to 3D. On the other hand, the treated NPL maintained its outstanding blue PL throughout the film for 60 minutes under water. After 60 min, it started to degrade but it still maintained blue PL up to 90 minutes (see Fig. 5g–j). Therefore, the fluoride passivated surface seems to prevent the immediate 2D to 3D phase transition in treated NPLs, maintaining blue PL in direct water contact for more than one hour. This result holds great promise for protecting the NPL from the moisture induced degradation.

#### 2.4.4. Stability against washing solvent

Next, we measured the optical stability of both the parent and treated NPL (*n* = 3) against mildly polar methyl acetate (MeAC) solvent, which is frequently used for washing of NCs/NPLs to remove excess capping ligands. For the parent NPL, the addition of increasing volumes of MeAC solvent resulted in structural conversion with higher thicknesses (*n* > 3), as confirmed by steady-state absorption and PL measurements (Fig. S10a of the ESI†). We observed the appearance of different red-shifted PL peaks, apart from the *n* = 3 peak, with the gradual addition of MeAC solvent. The PL maxima near 477 nm and 510 nm gradually appeared due to the formation of the *n* = 4 NPLs and bulk 3D NCs, respectively, after adding 20% (V/V) MeAC (see Fig. S9a of the ESI†). This observation indicates that the gradual addition of mildly polar MeAC solvent causes the loss of surface-bound oleylammonium capping ligands and halide ions.

On the other hand, with increasing vol% of MeAC solvent, the treated NPL does not show any change in excitonic absorption maxima or PL maxima (see Fig. S11b and c of the ESI†). The absence of any red-shifted PL peaks with the gradual addition of MeAC solvent rules out the possibility of the conversion of the *n* = 3 treated NPL structure into higher thickness structures (*n* > 3). This suggests a stable halide vacancy repaired surface with exceptional NPL surface–ligand interaction. Additionally, we observed an increase in PL intensity with the addition of up to 4.7 vol% MeAC, after which it gradually decreased (see Fig. S9c of the ESI†). This implies that MeAC washing could be a beneficial step for further increasing the PLQY of *n* = 3 treated NPLs. Furthermore, no change in the average lifetime was observed, indicating that the charge carrier dynamics remain unaffected by the MeAC solvent treatment (see Fig. S9d and S12 of the ESI†).

#### 2.4.5. Stability against ambient conditions

Finally, we evaluated the ambient stability of both parent and treated NPLs to demonstrate the efficacy of our modification. Preservation of NPLs under normal atmospheric conditions tends to accelerate their transformation into 3D NCs, as the loss of quantum confinement occurs through the protonation and deprotonation of dynamically bound capping ligands by converting oleylammonium ions into oleylamine.^16^

To assess crystalline stability, we recorded the PXRD patterns of both parent and treated NPLs (for *n* = 3 and 4) preserved in an open atmosphere. After 30 days, both of the treated NPLs maintained their crystallographic patterns and exhibited perfect blue PL under 365 nm UV excitation (see Fig. S10a and e of the ESI†). However, for both the parent NPLs, after 15 days, the films started showing green PL (see Fig. S10b and f of the ESI†). Also, two peaks near 15.0° and 30.3° appeared with higher intensity and their full width at half maximum (FWHM) became narrower compared to day 0 (see Fig. S10b and f of the ESI†). This result suggests the presence of the bulk 3D CsPbBr_3_ phase with larger crystal size, as indicated by the inverse relationship between the full width at half maximum (FWHM) of PXRD peaks and crystal size, as described by the popular Debye–Scherrer equation.^78^ Additionally, the characteristic low-angle reflection peaks for 2D stacking were partially (for *n* = 3 parent) or fully (for *n* = 4 parent) lost. This observation indicates the destruction of 2D stacking in the film after long-term exposure to an open atmosphere (see Fig. S10b and f of the ESI†). This phase transformation in the solid state is triggered by atmospheric conditions, particularly moisture and oxygen, as reported in previous studies.^24,79^ We also investigated the phase stability in the solution state for both parent and treated NPLs by measuring PL. Both the *n* = 3 and *n* = 4 treated NPLs exhibited excellent phase stability by showing intense deep blue PL over 30 days, maintaining their PL maxima at 453 nm and 470 nm, respectively, without the emergence of any new red-shifted peaks (see Fig. S10c and g of the ESI†). On the other hand, the PL maxima of both *n* = 3 and *n* = 4 parent NPLs slowly red shifted after 15 days, reaching 519 nm and 515 nm and green PL was observed for both samples (see Fig. S10d and h of the ESI†). These observations suggest impressive phase stability of 2D treated NPLs under ambient conditions, where surface fluorination and oleylammonium passivation simultaneously restrict the desorption of capping ligands.

#### 2.4.6. Plausible mechanism behind the enhanced stability against external threats

**Fig. 6:**
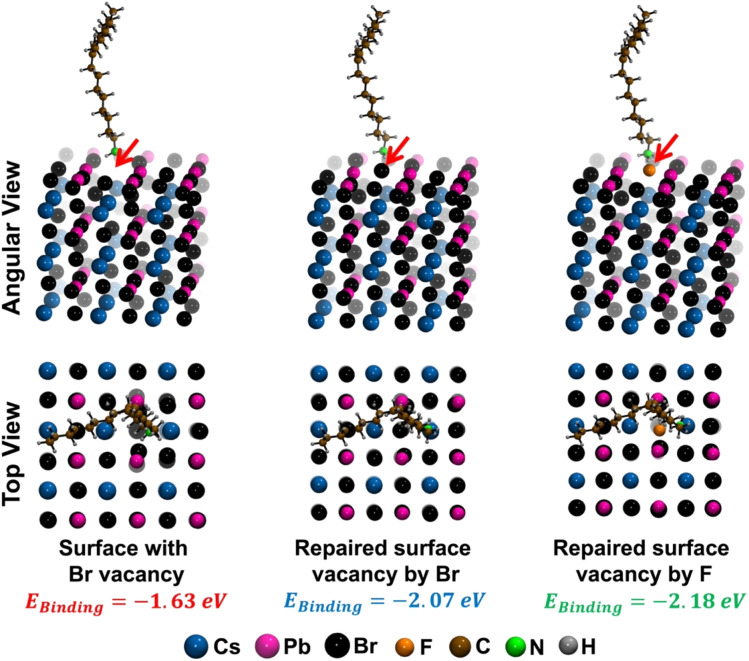
Angular and top views of optimized CsPbBr_3_ crystal slabs (100) with one Br vacancy (left panel), Br vacancy is filled by a bromine atom (middle panel) and Br vacancy is filled by a fluorine atom (right panel), in the presence of oleylammonium ligand.

The enhanced environmental stability of treated NPLs motivated us to investigate the stabilization energy associated with ligand binding (*E*_Binding_, see eqn (4)) between spacer oleylammonium and the NPL's surface. We performed density functional theory (DFT) calculations of the NPL system using VASP software (see detailed in the Experimental section). We modelled an *n* = 3 NPL system, featuring a defect-free PbBr_2_-terminated (100) facet. Next, we introduced a Br vacancy on the NPL surface as our parent NPL system typically suffers from Br vacancy related trap states (see Fig. 6). For the Br vacancy related surface, the calculated stabilization energy for ligand binding was found to be −1.63 eV. Upon filling the Br vacancy with a Br atom, the stabilization energy became more negative, measuring −2.07 eV. Instead of a Br atom, when we introduced an F atom into the vacancy, the stabilization energy upon ligand binding further reduced to −2.18 eV. This additional stabilization of the binding energy of 0.11 eV for F insertion in comparison to Br, indicates a significant improvement in ligand–surface interaction. This theoretical finding underscores the effectiveness of OAmF incorporation, providing deeper insight into the mechanisms responsible for increased stability.

**Scheme 3:**
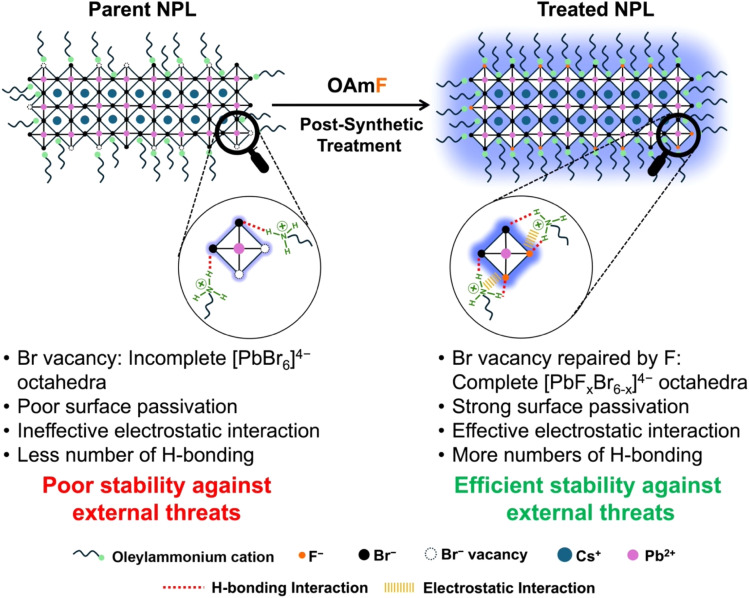
Plausible mechanism for the enhanced stability of treated NPLs against external perturbations like heat, UV irradiation, water and atmospheric conditions after post-synthetic treatment with oleylammonium fluoride (OAmF).

The parent NPLs typically exhibit significant surface defects, most notably bromide vacancies, which lead to an incomplete lead-bromide octahedral facet with a very low PLQY of <10%. This incomplete surface arrangement weakens the RP phase stabilizing interactions such as electrostatic and H-bonding interaction between the spacer oleylammonium and bromide ions, resulting in loosely bound spacers. Consequently, the poor surface organization of the parent NPLs in colloidal solution promotes their fusion into larger 3D NC aggregates *via* an Oswald ripening type mechanism, causing the breakdown of the 2D layered architecture under external perturbations like heat, light exposure, washing solvent and atmospheric conditions. Also, the presence of oleate anions on the surface may trigger the deprotonation of oleylammonium ions into oleylamine, which may accelerate this 2D to 3D phase transformation. After the OAmF post-synthetic treatment, all the previously mentioned challenges are effectively resolved up to an extent. The fluoride ions repair the bromide vacancies on the surface, while the synthesized oleylammonium cation form strong electrostatic and hydrogen-bonding interactions with both bromide and fluoride ions on the modified surface. Additionally, as proposed in an earlier report,^80^ the oleylammonium cation may occupy vacancies at the A-site, replacing Cs^+^ cations. This enhanced ligand binding with the modified [CsX] (X = F and Br) and [PbX_2_] (X = F and Br) terminated facets creates a robust and defect-free surface architecture that reinforces the stabilization of the Ruddlesden–Popper (RP) phase. This stable and defect-free surface, with strong coupling between the NPL surface and spacers, ensures the structural integrity needed to safeguard the 2D phase. It effectively prevents spacer loss and halide migration from the surface, shielding the material from various external perturbations. In Scheme 3, we have presented the plausible mechanism for the enhanced stability achieved through OAmF treatment.

## 3. Conclusions

In this report, we present a straightforward and cost-effective strategy to overcome the significant challenges associated with CsPbBr_3_ NPLs, specifically their poor optical properties and phase instability when exposed to various external perturbations. Our approach involves a post-synthetic treatment of CsPbBr_3_ NPLs with synthesized oleylammonium fluoride salts, representing a novel application in halide perovskite research for the first time according to the literature. The fluoride component of the salt effectively repairs bromide vacancy-related deep trap states, as evidenced by unprecedented improvements in photoluminescence quantum yield (PLQY) with detailed TRPL measurements. The fluoride rich surface interacts robustly with the primary oleylammonium cation *via* electrostatic interactions and hydrogen bonding, forming a protective shell around the treated NPL lattice. This stable fluoride-passivated facet restricts the loss of capping ligands, spacers and the migration of ions, thus preserving the 2D phase against external stresses such as heat, UV light, moisture, and oxygen. TRPL measurements under thermal stress provide further insights into the charge carrier dynamics and the existence of shallow trap states near the conduction band edge of the treated NPLs. Overall, our post-synthetic treatment elevates the photoluminescence quantum yield (PLQY) of blue-emitting CsPbBr_3_ NPLs to near unity while preserving their 2D phase stability against external exposures. This improvement will significantly extend their operational lifetime, making them highly suitable for future optoelectronic applications.

## 4. Experimental section

### 4.1. Materials

Lead bromide (99%, Sigma-Aldrich), cesium carbonate (99%, Sigma-Aldrich), oleic acid (TCI), oleyl amine (TCI), hydrofluoric acid, hexane (99.5%, HPLC, Finar Chemicals), toluene (99.5%, HPLC, RANKEM), diethyl ether (>99%, RANKEM), methyl acetate (>99%, RANKEM), acetone (99.5%, HPLC, Finar Chemicals), ethanol (99.9%, Changshu Hongsheng Fine Chemicals Co., Ltd) was used as received.

### 4.2. Synthesis of parent CsPbBr_3_ nanoplates (NPLs) (*n* = 3 and *n* = 4)

To prepare CPB NPLs of specific thickness, we followed the synthesis procedure reported by Bohn *et al.*^18^ 0.1 mmol of cesium carbonate was reacted with 10 mL of oleic acid at 100 °C for 1 hour and used as a Cs-oleate stock solution. Next, 0.1 mmol of PbBr_2_ was taken with 100 μL of oleic acid and 100 μL of oleyl amine in 10 mL of toluene and then reacted for 1 hour at 100 °C until the solution became clear. This solution was used as the PbBr_2_ precursor solution. For the preparation of NPLs with thicknesses of *n* = 3 and *n* = 4, 1.5 mL and 1.2 mL of the PbBr_2_ precursor solution were taken respectively in glass vials with magnetic beads under normal room temperature conditions. After this, we followed the same procedure for both cases. Then, into the vigorously stirred PbBr_2_ precursor solution, 150 μL of Cs-oleate stock solution was added and stirred for 5–10 seconds, followed by the addition of 2 mL of acetone to trigger the formation of NPLs. Then it was centrifuged at 3000 rpm for 3 minutes and the supernatant was discarded. Then, we added 2 mL of hexane into the residual solution and used it as the parent NPL. As hexane is a completely non-polar solvent having a lower dielectric constant of 1.88 compared to toluene (2.38), the NPL remained more stable in hexane, and that's why all measurements were performed in hexane. For all optical measurements, we took 100 μL of parent NPL solution and diluted it with 2 mL of hexane (D20). Only for temperature-dependent purposes, we used toluene instead of hexane.

### 4.3. Synthesis of oleylammonium fluoride (OAmF)

In a 50 mL round bottom flask, we took 0.22 mmol of oleyl amine (7.2 mL) with 30 mL of ethanol as a solvent, and the temperature was maintained at 0–5 °C. Then, we added 0.22 mmol of hydrofluoric acid (0.8 mL) dropwise and stirred for 3 hours. Then, we evaporated the solvent using a rotary evaporator and washed the white solid powder with diethyl ether (10 times). The fine white powder was preserved as OAmF salt for further use and characterized by NMR (^1^H, ^13^C, and ^19^F-NMR) and IR spectroscopy (see Fig. S12 of the ESI†).

### 4.4. Preparation of oleylammonium fluoride (OAmF) stock solution and post-synthetic treatment of CsPbBr_3_ nanoplates with OAmF

To prepare the OAmF stock solution, 5.6 mg of OAmF powder was added to 3.5 mL of hexane and heated gently to dissolve the same. The concentration of OAmF stock solution was ∼5.6 mM. For the post-synthetic treatment, 100 μL of parent NPL solution was diluted with 2 mL of hexane, and an appropriate volume of OAmF stock solution was added at room temperature and stirred for 30 minutes. We carefully optimized the needed volume of OAmF stock solution, which was found to be 20 μL and 15 μL for *n* = 3 and *n* = 4 CPB NPLs, respectively.

### 4.5. Femtosecond fluorescence up-conversion measurements

Femtosecond time-resolved fluorescence was measured through the fluorescence up-conversion method on a commercial setup (FOG-100, CDP Corp. Russia). The details of this setup are available in our earlier publications.^81–84^ Briefly, the 800 nm output from a mode-locked Ti:sapphire oscillator with a repetition rate of 80 MHz (Mai Tai HP, Spectra Physics, USA) was frequency-doubled using a 0.2 mm β-barium borate crystal (BBO, *θ* = 25° and *φ* = 90°). This 400 nm light was used to excite the sample taken in a rotating sample cell positioned under the magic angle condition with respect to the fundamental light. The pump power was maintained to be 10 mW. The fluorescence emitted from the sample was then mixed with 800 nm fundamental light on a 0.5 mm BBO crystal (*θ* = 38° and *φ* = 90°) to generate the sum frequency light, which was then dispersed by a monochromator and detected with a photomultiplier tube. The instrument response function (IRF) was obtained by upconverting the Raman signal from the solvent and it was found to be Gaussian in nature having a full width at half maxima of 300 fs. The recorded fluorescence transients were deconvoluted using the measured IRF with IgorPro software.

### 4.6. Steady-state and nanosecond time-resolved spectroscopy measurement

Absorption and photoluminescence (PL) spectra were recorded on a commercial UV-visible spectrophotometer (UV-2450, Shimadzu, Japan) and a spectrofluorimeter (FluoroMax4, Jobin-Yvon, USA), respectively. All samples were excited at 350 nm. For temperature-dependent PL measurements, we controlled the temperature by using an in-built temperature controller with 0.5 °C accuracy. The nanosecond PL lifetime was measured using a time-correlated single photon counting (TCSPC) (LifeSpecII, Edinburgh Instruments, UK) setup under magic angle polarization reported in our previously published articles.^85,86^ In brief, the instrument response function (IRF) of the setup was measured to be ∼190 ps. In all lifetime measurements, the sample was excited with a 405 nm diode laser (EPL-405, Edinburgh Instruments, UK) at its emission maximum. The deconvolution of the PL decay was performed using FAST software and the average lifetime (*τ*_avg_) was calculated using the following equation2
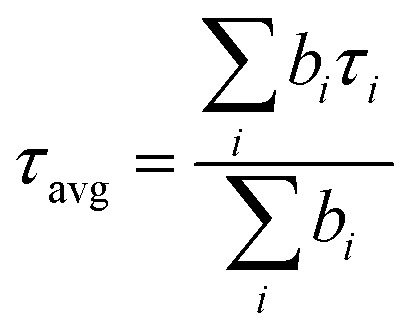
where *b*_*i*_ is the amplitude associated with the *i*-th lifetime component (*τ*_*i*_).

### 4.7. Photoluminescence quantum yield (PLQY) measurement

The PLQY of the sample was measured using an integrating-sphere (Quanta-Φ, HORIBA Scientific, Japan) using eqn (3).3
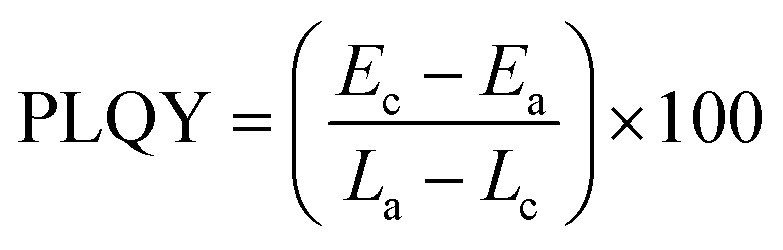


In the above equation, *E*_c_ and *L*_c_ are the PL intensity and scattering of the sample and *E*_a_ and *L*_a_ are the PL intensity and scattering of the blank, respectively.

### 4.8. Materials characterization techniques

The PXRD measurement was performed using a PANalytical Xpert powder diffractometer. The source of incident radiation was Cu-Kα (0.154 nm) and the diffraction angle (2*θ*) range was from 3° to 50° with a step size of 0.01°. The hexane dispersion of both parent and treated NPLs (both *n* = 3 and *n* = 4) was drop-cast on a clean glass side and left in an open atmosphere for complete evaporation of the solvent. SAXS measurements were performed using a PANalytical Empyrean set up with a diffraction angle range of 1.5°–10° and a step size of 0.01°. The HRTEM images were collected using a Titan G2 60-300 instrument. A sufficiently diluted solution of both parent and treated NPLs (both *n* = 3 and *n* = 4) was drop-cast on a Cu mesh 300 grid (Ted Pella) and dried overnight to capture the images. XPS measurements were performed using a PHI 5000 Versa Prob II model. The sample was drop-cast on a very thin coverslip and dried in open air. The FT-IR spectroscopy measurements were performed by using a Bruker Alpha-P FTIR by drop casting the sample on a KBr pellet. ^1^H-NMR, ^13^C-NMR and ^19^F-NMR spectra were recorded on a JEOL 500 MHz NMR instrument with CDCl_3_ as the locking solvent.

### 4.9. Computational methods

The Vienna *Ab initio* Program Software Package (VASP) was used to perform spin polarized DFT calculations of the *n* = 3 RP NPL before and after treatment.^87–89^ We created an *n* = 3 RP NPL and introduced a vacuum of ∼13 Å along the cleaved direction to prevent any interaction between the two subsequent periodic images. The exchange-correlation functional was obtained from the Perdew–Burke–Ernzerhof formulation.^89^ The projector-augmented-wave (PAW) method was used to treat the core electrons of different atoms. For F and Br atoms, 7 electrons were taken as core electrons, whereas, for Cs, Pb, N, C, and H atoms, 9, 14, 5, 4, and 1 electrons were taken as core electrons, respectively.^90^ Dispersion correction was incorporated using the Grimme D3 method.^91^ Initially, a high-plane wave energy cut-off of 600 eV was used for unit cell optimization, and later, we used a cut-off of 500 eV for all production calculations. The initial structural configuration was obtained from the crystallographic database of cubic CsPbBr_3_ crystals.^92^ The calculations were allowed to run until an energy convergence criterion of 10^−6^ eV was reached. The preliminary structure of CsPbBr_3_ with the (100) facet was obtained with the help of a Python program developed by Sun and Ceder.^93^ The binding energy of the ligand with the NPL surface was calculated using^45^

4 *E*_Binding_ = *E*_Tot_ − *E*_V_ − *E*_Ligand_

where *E*_Tot_ is the energy of the NPL, which is completely passivated by the ligand, *E*_V_ is the energy of the NPL with one Br vacancy on the surface, and *E*_Ligand_ is the energy of the ligand.

## Data availability

All data will be available on request from AS (arghyas@iitk.ac.in) or PS (psen@iitk.ac.in).

## Author contributions

The experiment was conceptualized by AS and PS. AD performed fluorescence up-conversion measurements and the corresponding data fitting. AS performed all data plotting and wrote the initial draft. AD and ALB contributed in the theoretical calculations. PS supervised the work, provided valuable insights through discussion, acquired funding, and reviewed and finalized the manuscript.

## Conflicts of interest

The authors declare no competing financial interest.

## Supplementary Material

SC-OLF-D4SC05565A-s001
