# Supplementary material for: Oleylammonium fluoride passivated blue-emitting 2D CsPbBr3 nanoplates with near-unity photoluminescence quantum yield: safeguarding against threats from external perturbations
Source: Chem Sci. 2024 Nov 26;16(2):735–52. doi: 10.1039/d4sc05565a (PMC11610764; doi:10.1039/d4sc05565a)
Supplement: SC-016-D4SC05565A-s001 [file SC-016-D4SC05565A-s001.pdf]

Supplementary Information (SI) for Chemical Science.
This journal is © The Royal Society of Chemistry 2024

Electronic Supplementary Information (ESI)

for

Oleylammonium Fluoride Passivated Blue-Emitting 2D $CsPbBr_3$ Nanoplates with Near-Unity Photoluminescence Quantum Yield: Safeguarding against Threats by External Perturbations

Arghya Sen,[1] Abhijit Dutta,[1] Abir Lal Bose[2] and Pratik Sen[1,*]

[1]Department of Chemistry, Indian Institute of Technology Kanpur, Kanpur – 208 016, UP, India

[2]Department of Chemical Engineering, Indian Institute of Technology Kanpur, Kanpur – 208 016, UP, India

*Corresponding author; E-mail: psen@iitk.ac.in

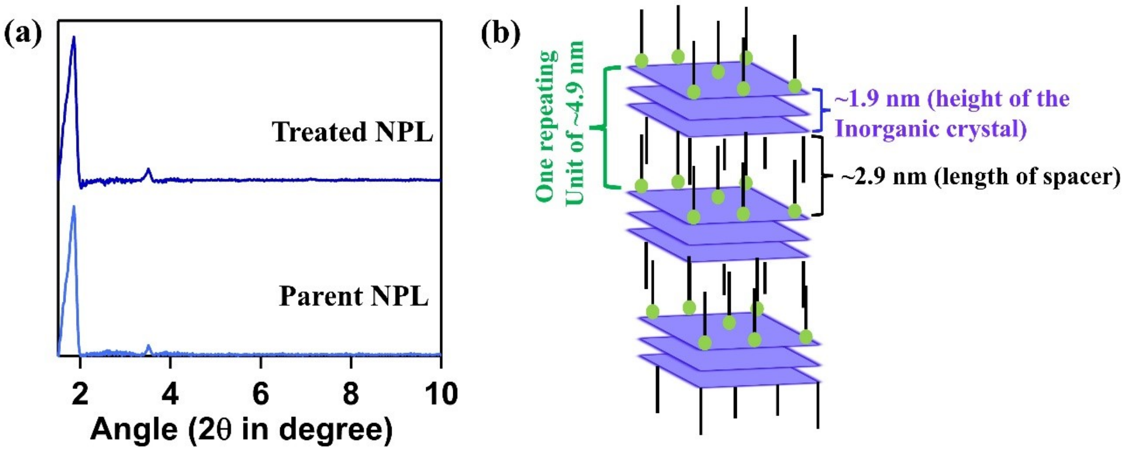

Figure S1. (a) Small angle X-ray scattering (SAXS) measurement of $n = 3$ parent and treated NPL. (b) Structural representation of colloidal Ruddlesden-Popper (RP) type $n = 3$ CsPbBr3 NPL in solution with applicable distance of inorganic layer and spacer ligands.

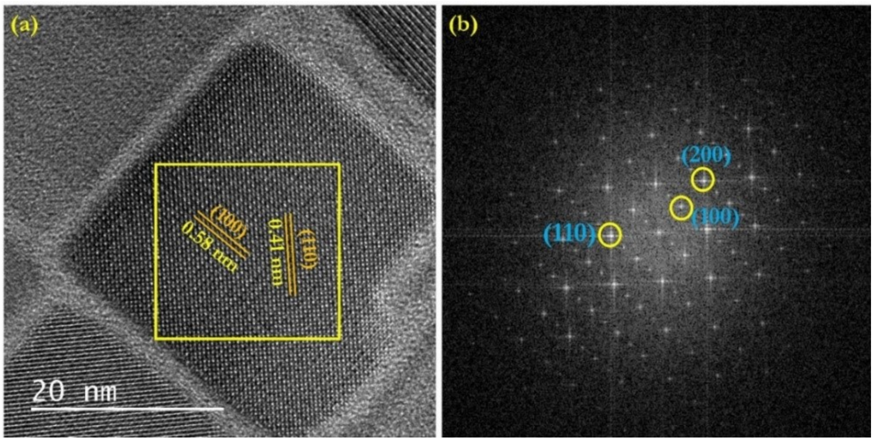

Figure S2. (a) High-resolution TEM image of parent $CsPbBr_3$ NPL (n = 3) showing (100) and (110) crystallographic planes having an interplanar distance of ~0.58 nm and ~0.41 nm. (b) The Fast Fourier transformed (FFT) image obtained from the selected area of Figure (a) showing crystallographic planes (100), (200), and (110).

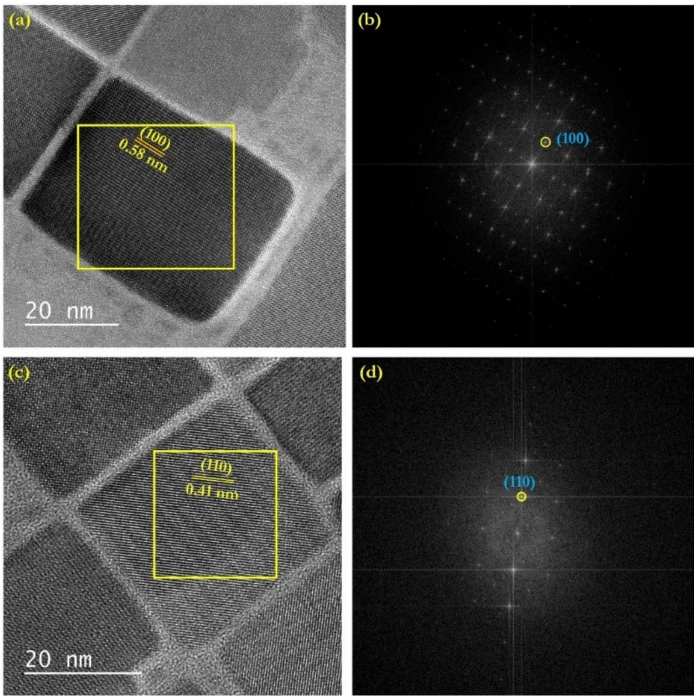

Figure S3. (a) and (c) High-resolution TEM images of the treated $CsPbBr_3$ NPL ($n = 3$) showing (100) and (110) crystallographic planes having an interplanar distance of ~0.58 nm ~0.41 nm, respectively. (b) The FFT image obtained from the selected area of figure a showing the crystallographic plane (100) (d) The FFT image obtained from the selected area of figure b showing the crystallographic plane (110).

Table S1. The binding energy values for F-1s, Cs-3d, Pb-4f, and Br-3d for N3 parent and treated RP NPL.

Elements	Parent NPL Binding Energy (eV)	Treated NPL Binding Energy (eV)
F	-	685.6
N	401.8	402.2
Cs	724.2, 738.1	724.5, 738.4
Pb	138.2, 143.1	138.5, 143.4
Br	68.06, 69.05	68.29, 69.27

Supporting Note S1.

The parent NPL ($n = 4$) shows two lower angle reflections at 3.3° and 5.0° for (00l) staking with two characteristic peaks near 15.0° (100) and 30.4° (200) for the cubic $CsPbBr_3$ crystal structure. After treatment with OAmF, we did not observe any characteristic change in the peak position, which confirmed the unaffected crystal structure (see figure S3a).

For N4 parent NPL, we obtained predominantly cubic/rectangular morphology, which converted mostly square shape after treatment (see figures S3b-c). Also, the HAADF images of the parent and treated N4 NPL confirm the high crystallinity of the NPL (see figures S3b-c). From the HRTEM image, we isolated (110) crystallographic plane with an interplanar distance of ~0.41 nm (see figures S4a-c). On the other hand, we isolated (200) crystallographic planes with an interplanar distance of ~0.29 nm for N4-treated NPL (see figures S4d-f). From the elemental mapping, we obtained the characteristic count of the F element along with Cs, Pb, and Br elements, confirming the surface passivation by OAmF ligands as well (see figures S3d-h).

Next, from the XPS, we obtained the signal of F element at 685.06 eV refers to the presence of F^- ions in the surface of the N4 treated NPL (see figure S3i). Also, after deconvolution, we obtained characteristic N-1s, Cs-3d, Pb-4f, and Br-3d peaks for both parent and treated N4 NPL whereas the shift binding energy towards higher value for every element justifies the insertion of F-atom on the surface like our discussion for N3 case (see figures S3j-m and table S2). These observations refer to concrete surface architecture with strong ligand-NPL surface interaction and the formation of surface Pb-F bonds like N3 NPL.

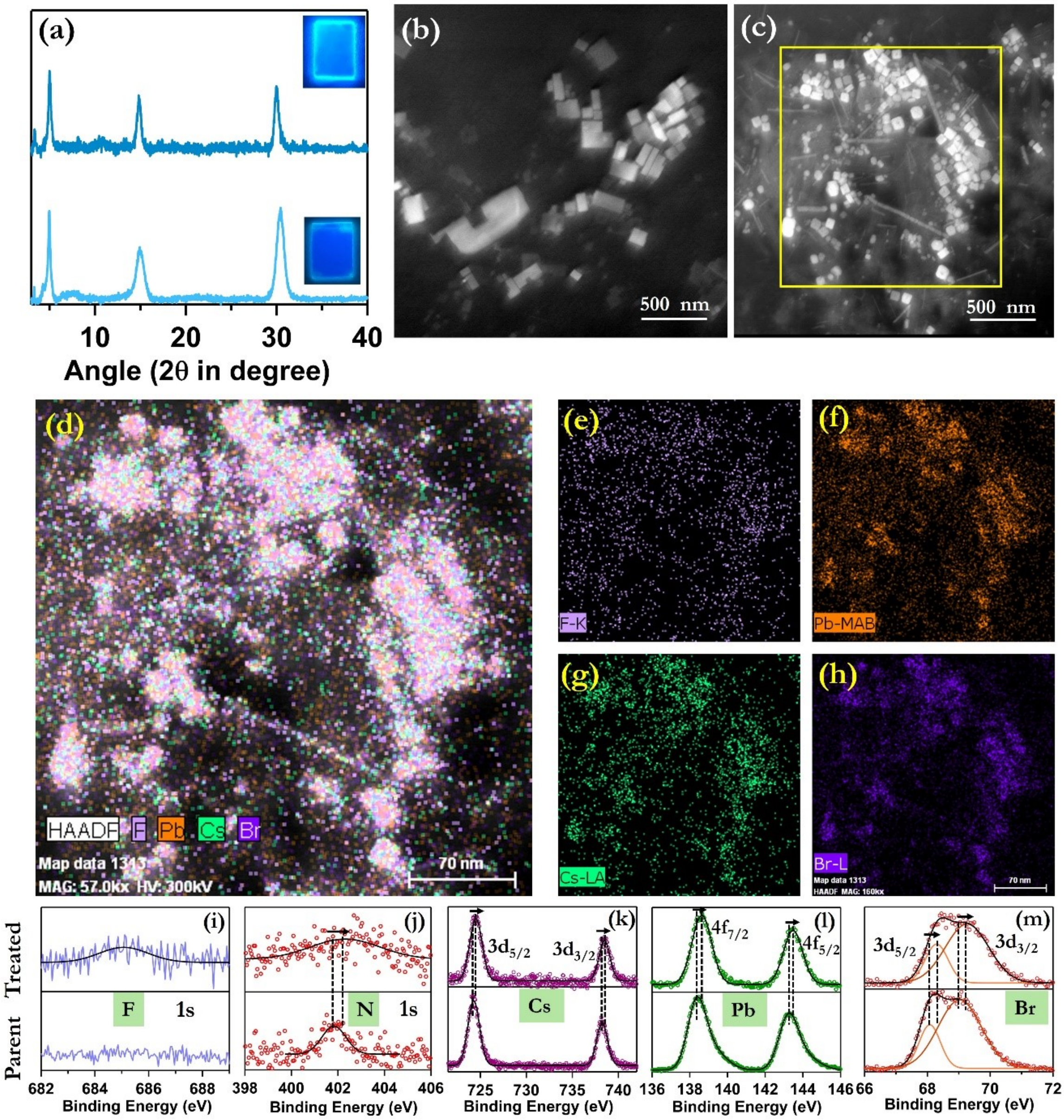

Figure S4. (a) PXRD pattern of the parent (lower panel) and treated (upper panel) $CsPbBr_3$ NPL ($n = 4$). The inset images represent the PL of parent NPL film (lower panel) and treated NPL film (upper panel) under 365 nm UV excitation. The high-angle annular dark field (HAADF) imaging of the (b) parent NPL ($n = 4$) and (c) treated NPL ($n = 4$).(d) The overall elemental mapping from the selected portion of figure c shows the presence of Cs, Pb, Br, and F elements throughout the NPL. The individual elemental mapping of (e) F (f) Pb (g) Cs and (h) Br elements. The high-resolution XPS of the parent (lower panel) and treated (upper panel) of $CsPbBr_3$ NPL ($n = 4$) of (i) F-1s (j) N-1s, (k) Cs-3d, (l) Pb-4f and (m) Br-3d elements.

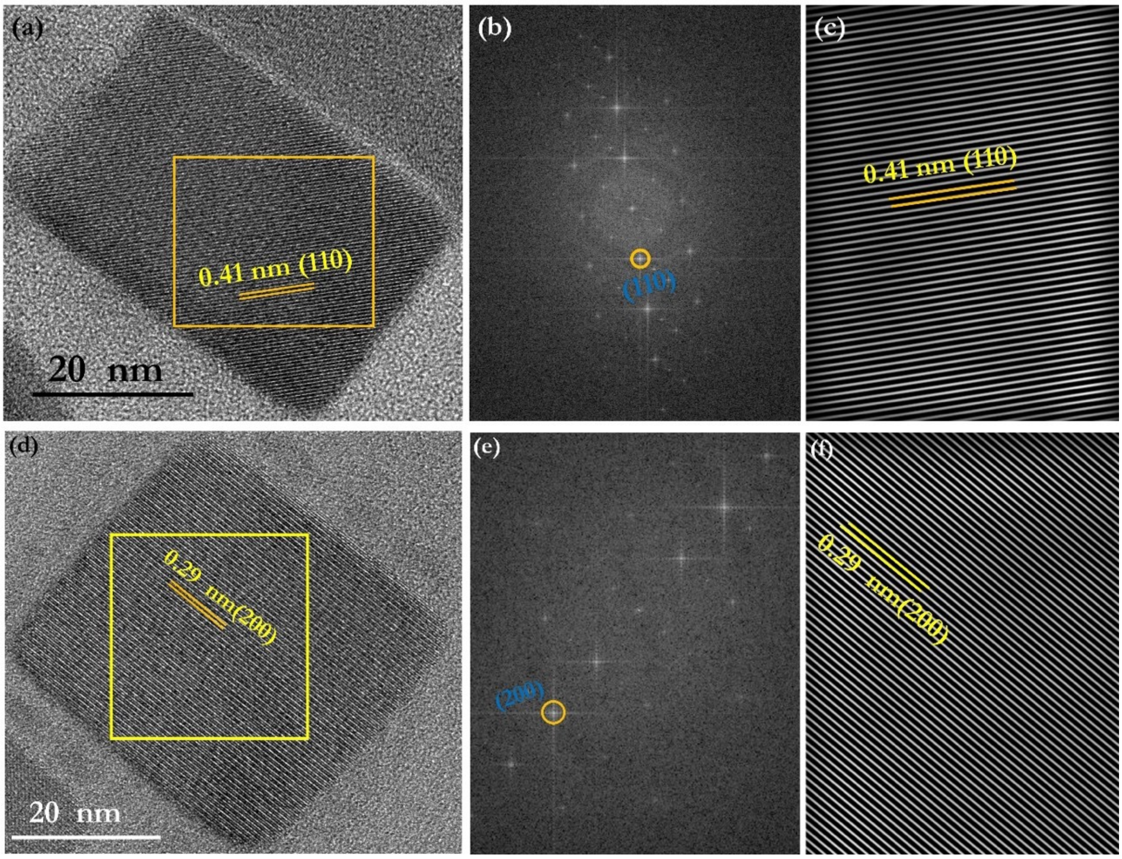

Figure S5. (a) HRTEM image of parent $CsPbBr_3$ NPL ($n = 4$) showing (110) crystallographic plane having an interplanar distance of ~0.41nm. (b) The Fast Fourier transformed (FFT) image obtained from the selected area of Figure (a) showing the crystallographic plane (110). (c) Fourier filtered image from figure b showing crystallographic plane (110) having an interplanar distance of ~0.41 nm (d) HRTEM image of treated $CsPbBr_3$ NPL ($n = 4$) showing (200) crystallographic plane having an interplanar distance of ~0.29 nm. (e) The Fast Fourier transformed (FFT) image obtained from the selected area of figure d showing the crystallographic plane (200). (f) Fourier filtered image from figure e showing crystallographic plane (200) having an interplanar distance of ~0.29 nm.

Table S2. The binding energy values for N-1s, Pb-4f, and I-3d photoelectrons for N4 parent and treated RP NPL.

Elements	Parent NPL Binding Energy (eV)	Treated NPL Binding Energy (eV)
F	-	685.06
N	401.8	402.3
Cs	724.27, 738.2	724.48, 738.49
Pb	138.36, 143.23	138.63, 143.47
Br	68.06, 69.02	68.28, 69.26

Supporting Note S2:

We have determined the radiative rate constant (k_r) and the non-radiative rate constant (k_{nr}) by using the following methods:

As we know,

$$PLQY = \frac{k_r}{k_r + k_{nr}} \dots (1)$$

and

$$\tau_{av} = \frac{1}{k_r + k_{nr}} \dots (2)$$

Now, by using equation (1) and (2), we can write that

$$k_{rad} = \frac{PLQY}{\tau_{av}} \dots (3)$$

and

$$k_{nr} = \frac{1 - PLQY}{\tau_{av}} \dots (4)$$

Table S3. Change of the excitonic peak (nm) and PL Max (nm) for $CsPbBr_3$ NPL $(n = 3)$ with gradual addition of OAmF stock solution.

Sample	Excitonic Peak (nm)	PL Max (nm)
Parent NPL	441.4	453
Parent NPL + 5 µL	441.4	453
Parent NPL + 10 µL	441.4	453
Parent NPL + 15 µL	441.4	453
Parent NPL + 20 µL	441.4	453
Parent NPL + 25 µL	442.4	454

Table S4. Change of the excitonic peak (nm) and PL Max (nm) for $CsPbBr_3$ NPL $(n = 4)$ with the gradual addition of OAmF stock solution.

Sample	Excitonic Peak (nm)	PL Max (nm)
Parent NPL	459.8	470
Parent NPL + 5 µL	459.8	470
Parent NPL + 10 µL	459.8	470
Parent NPL + 15 µL	459.8	470
Parent NPL + 20 µL	461.6	470
Parent NPL + 25 µL	461.8	471

Table S5. PL decay parameters of $CsPbBr_3$ NPL $(n = 3)$ with the gradual addition of OAmF stock solution. The samples were excited by 375 nm pulsed diode LASER

Sample	$\tau_1(ps)$	$B_1(\%)$	$\tau_2(ns)$	$B_2(\%)$	$\tau_3(ns)$	$B_3(\%)$	$\tau_{av}(ns)$
Parent NPL	210	0.34 (66.7)	1.1	0.14 (27.4)	3.3	0.03 (5.9)	0.63
Parent NPL + 5 μL	385	0.2 (48.7)	1.8	0.17 (41.4)	4.4	0.04 (9.9)	1.4
Parent NPL + 10 μL	422	0.12 (30.7)	2.3	0.17 (43.5)	5.3	0.1 (25.8)	2.5
Parent NPL + 15 μL			2.1	0.10 (30.3)	5.4	0.23 (69.7)	4.4
Parent NPL + 20 μL			2.2	0.11 (33.3)	5.5	0.22 (66.7)	4.4
Parent NPL + 25 μL			2.2	0.10 (31.2)	5.6	0.22 (68.8)	4.5

Table S6. PL decay parameters of $CsPbBr_3$ NPL $(n = 4)$ with the gradual addition of OAmF stock solution. The samples were excited by 375 nm pulsed diode LASER.

Sample	$\tau_1(ps)$	$B_1(\%)$	$\tau_2(ns)$	$B_2(\%)$	$\tau_3(ns)$	$B_3(\%)$	$\tau_{av}(ns)$
Parent NPL	340	0.2 (50)	1.8	0.15 (37.5)	4.7	0.05 (12.5)	1.4
Parent NPL + 5 μL	424	0.1 (28.5)	2.6	0.15 (42.8)	5.9	0.1 (28.7)	2.9
Parent NPL + 10 μL			2.0	0.1 (34.5)	5.6	0.19 (65.5)	4.3
Parent NPL + 15 μL			2.2	0.1 (34.5)	5.7	0.19 (65.5)	4.5
Parent NPL + 20 μL			1.9	0.08 (27.5)	5.5	0.21 (72.5)	4.5

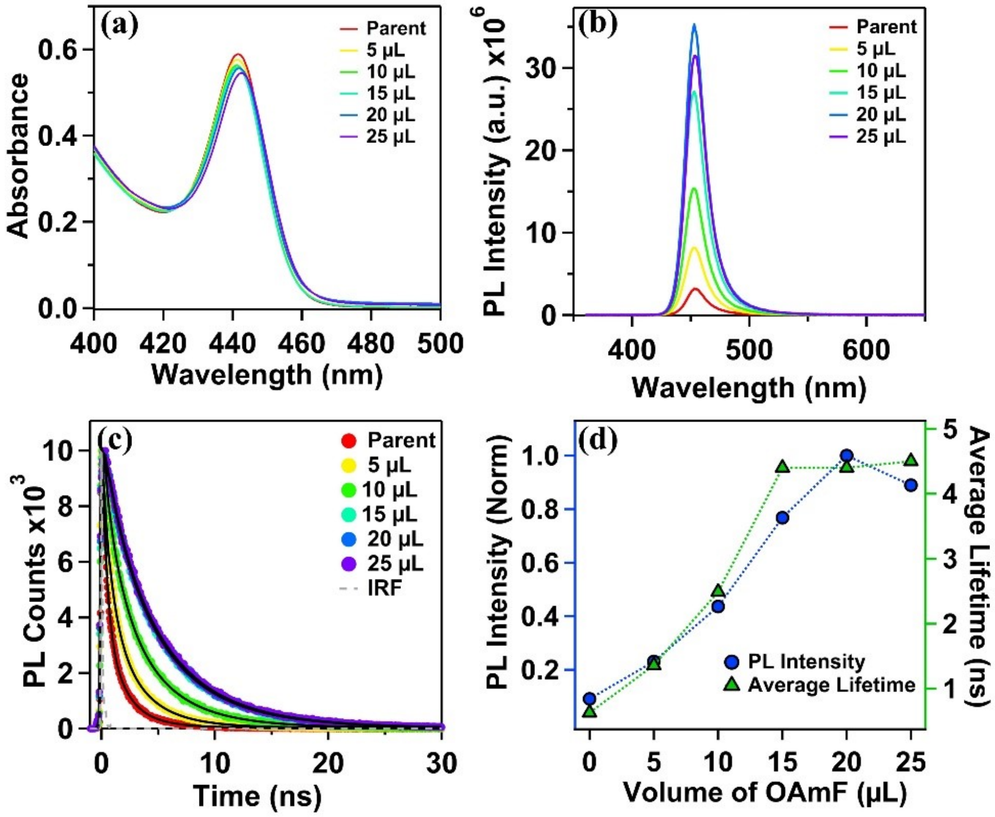

Figure S6. The change of the optical properties of $CsPbBr_3$ NPL (n = 3) with the gradual addition of OAmF stock solution. Change in (a) absorption spectra, (b) PL spectra, (c) PL transients, and (d) PL intensity (normalized at PL maxima) and average lifetime (in ns). All PL spectra were recorded by exciting the sample at 350 nm excitation. The nanosecond PL transients were recorded by exciting the sample with 375 nm pulsed diode LASER.

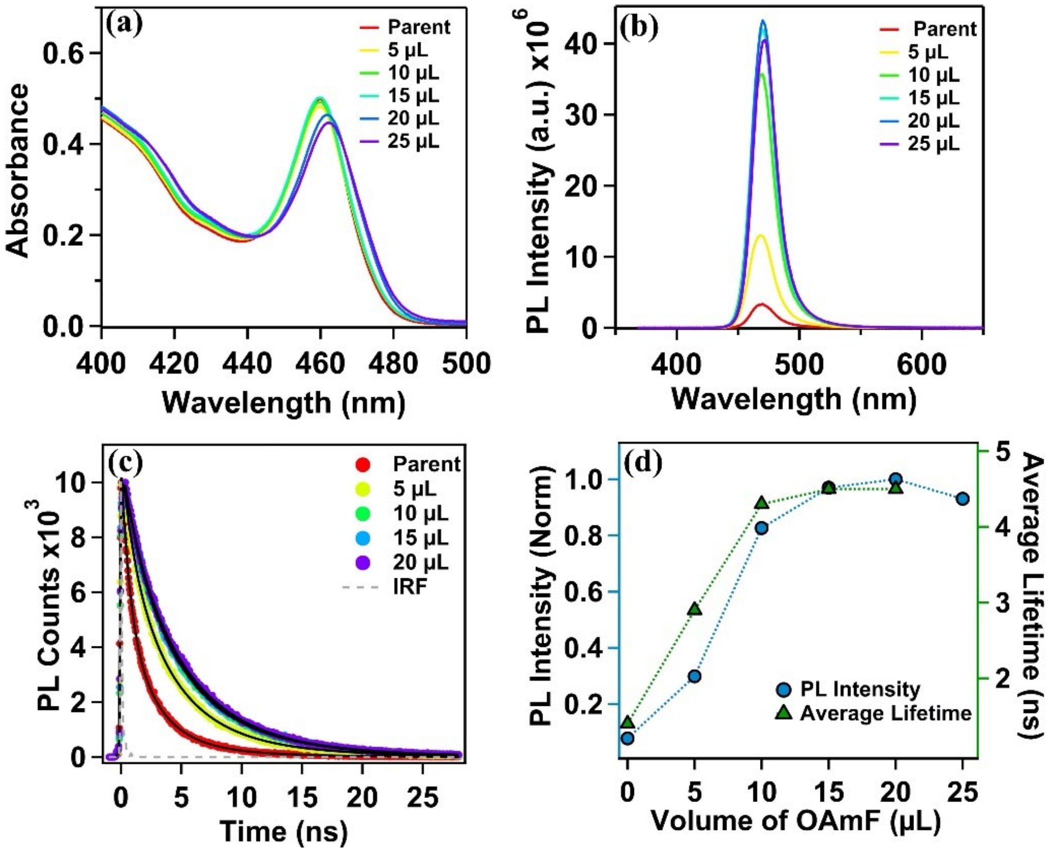

Figure S7. The change of the optical properties of $CsPbBr_3$ NPL (n = 4) with the gradual addition of OAmF stock solution. Change in (a) absorption spectra, (b) PL spectra, (c) PL transients, and (d) PL intensity (normalized at PL maxima) and average lifetime (in ns). All PL spectra were recorded by exciting the sample at 350 nm excitation. The nanosecond PL transients were recorded by exciting the sample with 375 nm pulsed diode LASER.

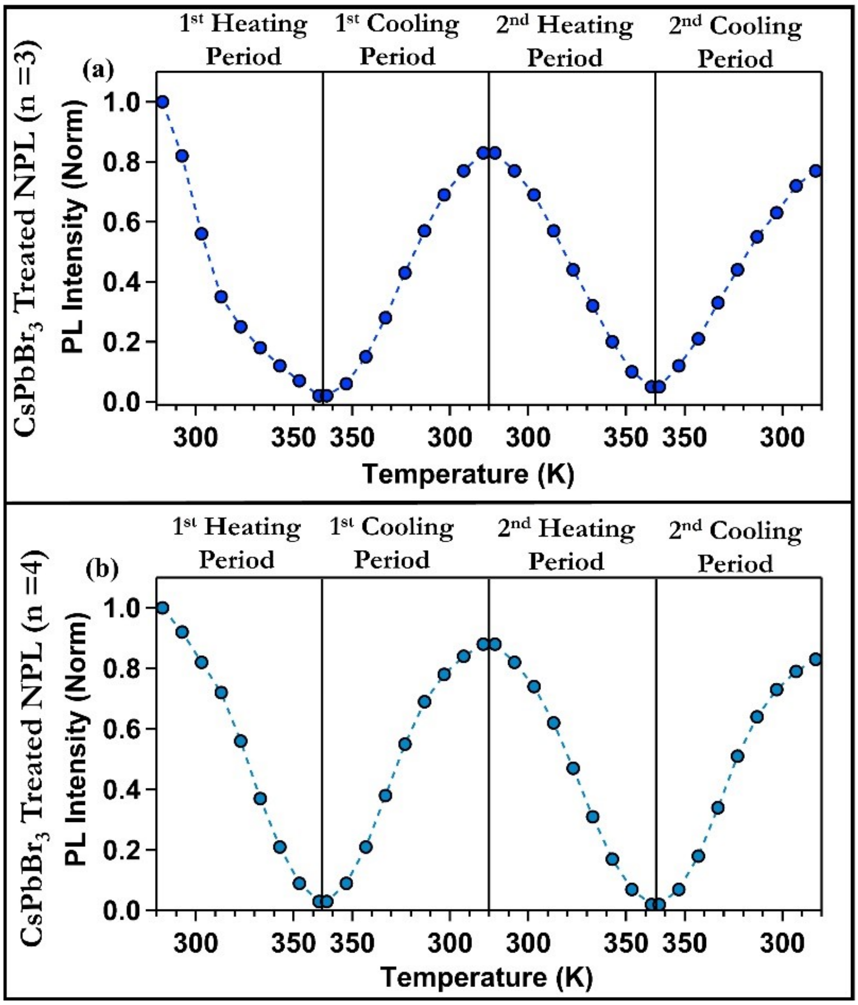

Figure S8. The change of normalized PL intensity (at PL maxima) of the (a) treated $CsPbBr_3$ NPL ($n = 3$) and (b) treated $CsPbBr_3$ NPL ($n = 4$) with increasing temperature (1st heating cycle), decreasing temperature (1st cooling cycle), increasing temperature (2nd heating cycle), and decreasing temperature (2nd cooling cycle).

Table S7. PL decay parameters of OAmF treated $CsPbBr_3$ NPL ($n = 3$) for 1st heating period (283K to 363K) at 10K intervals. The samples were excited by 375 nm pulsed diode LASER.

**Temperature (K)**	$\tau_1(ps)$	**B_1(%)**	$\tau_2(ns)$	**B_2(%)**	$\tau_3(ns)$	**B_3(%)**	$\tau_{av}(ns)$
283			2.0	0.14 (43.8)	4.3	0.18 (56.2)	3.3
293			2.2	0.14 (43.7)	4.7	0.18 (56.3)	3.6
303			2.0	0.11 (34.4)	4.8	0.21 (65.6)	3.8
313			2.2	0.11 (35.4)	5.2	0.20 (64.6)	4.1

323			2.1	0.11 (34.3)	5.4	0.21 (65.7)	4.3
333			1.8	0.11 (34.3)	5.4	0.21 (65.7)	4.2
343			1.6	0.13 (40.6)	5.4	0.19 (59.4)	3.8
353	289	0.15 (37.5)	1.9	0.13 (32.5)	5.5	0.12 (30)	2.4
363	200	0.24 (52.1)	1.4	0.14 (30.4)	5.0	0.09 (17.5)	1.5

Table S8. PL decay parameters of OAmF treated $CsPbBr_3$ NPL ($n = 3$) for 1st cooling period (363K to 283K) at 10K intervals. The samples were excited by 375 nm pulsed diode LASER.

**Temperature (K)**	$\tau_1(ps)$	**B_1(%)**	$\tau_2(ns)$	**B_2(%)**	$\tau_3(ns)$	**B_3(%)**	$\tau_{av}(ns)$
363	200	0.24 (52.1)	1.4	0.14 (30.4)	5.0	0.09 (17.5)	1.5
353	231	0.13 (31.7)	1.9	0.13 (31.7)	5.6	0.15 (36.6)	2.7
343			1.5	0.11 (34.3)	5.5	0.21 (65.7)	4.1
333			2.0	0.11 (34.3)	5.6	0.21 (65.7)	4.4
323			2.0	0.10 (32.2)	5.4	0.21 (67.8)	4.3
313			1.7	0.10 (31.2)	5.0	0.22 (68.8)	4.0
303			1.8	0.11 (34.3)	4.7	0.21 (65.7)	3.7
293			1.9	0.13 (40.6)	4.5	0.19 (59.4)	3.4
283			2.0	0.16 (48.4)	4.3	0.17 (51.6)	3.2

Table S9. PL decay parameters of OAmF treated $CsPbBr_3$ NPL ($n = 3$) for 2nd heating period (283K to 363K) at 10K intervals. The samples were excited by 375 nm pulsed diode LASER.

Temperature (K)	$\tau_1(ps)$	B1(%)	$\tau_2(ns)$	B2(%)	$\tau_3(ns)$	B3(%)	$\tau_{av}(ns)$
283			2.0	0.16 (48.4)	4.3	0.17 (51.6)	3.2
293			2.1	0.15 (46.8)	4.7	0.17 (53.2)	3.5
303			2.1	0.12 (38.7)	4.9	0.19 (61.3)	3.8
313			2.1	0.11 (34.3)	5.1	0.21 (65.7)	4.1
323			1.7	0.10 (31.2)	5.3	0.22 (68.8)	4.2
333			1.9	0.11 (34.3)	5.5	0.21 (65.7)	4.3
343			1.7	0.13 (39.3)	5.4	0.20 (60.7)	3.9
353	242	0.15 (35.7)	1.9	0.13 (30.9)	5.4	0.14 (33.4)	2.5
363	204	0.23 (47.9)	1.6	0.15 (31.2)	5.2	0.10 (20.9)	1.7

Table S10. PL decay parameters of OAmF treated $CsPbBr_3$ NPL ($n = 3$) for 2nd cooling period (363K to 283K) at 10K intervals. The samples were excited by 375 nm pulsed diode LASER.

Temperature (K)	$\tau_1(ps)$	B1(%)	$\tau_2(ns)$	B2(%)	$\tau_3(ns)$	B3(%)	$\tau_{av}(ns)$
363	204	0.23 (47.9)	1.6	0.15 (31.2)	5.2	0.10 (20.9)	1.7
353	311	0.12 (30.7)	2.3	0.14 (35.8)	6.1	0.13 (33.5)	2.9
343			1.5	0.14 (35.4)	5.5	0.20 (64.6)	3.8
333			1.6	0.11 (34.3)	5.5	0.21 (65.7)	4.1
323			1.9	0.11 (34.3)	5.3	0.21 (65.7)	4.1
313			2.1	0.12 (38.7)	5.2	0.19 (61.3)	4.0
303			2.0	0.12 (38.7)	4.8	0.19 (61.3)	3.7
293			1.9	0.13 (40.6)	4.4	0.19	3.4

				(59.4)	
283	2.0	0.16 (50.0)	4.3	0.16 (50.0)	3.1

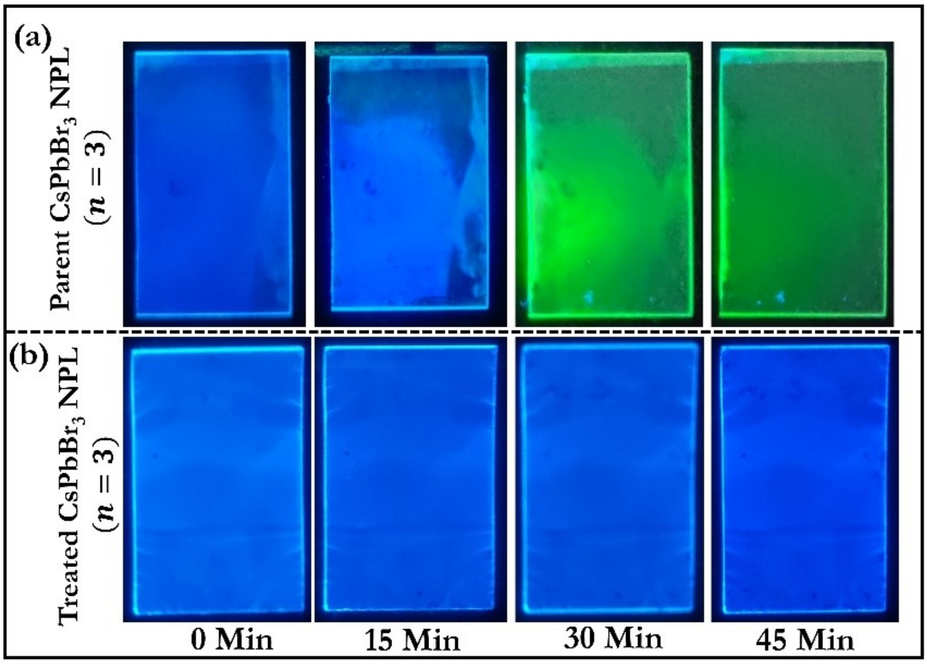

Figure S9. The heat stability of the (a) parent and (b) treated $CsPbBr_3$ NPL's $(n = 3)$ film at 343K. The photographic images represent the PL of the film under 365 nm UV excitation captured at different times i.e., Min-0, Min-15, Min-30, and Min-45 with heating.

Table S11. The PL decay parameters of treated NPL before and after UV irradiation.

**Sample**	$\tau_1(ns)$	B_1**(%)**	$\tau_2(ns)$	B_2**(%)**	$\tau_{av}(ns)$
Treated NPL at 0 Hour $(n = 3)$	2.2	0.10 (31.2)	5.6	0.22 (68.8)	4.5
Treated NPL after 5 hours $(n = 3)$	2.2	0.10 (30.3)	5.5	0.23 (69.7)	4.5
N4 Treated NPL at 0 Hour $(n = 4)$	2.2	0.10 (34.5)	5.7	0.19 (65.5)	4.5
N4 Treated NPL after 5 hours $(n = 4)$	2.2	0.10 (30.3)	5.6	0.23 (69.7)	4.6

Table S12. The PL decay parameters of treated NPL $(n = 3)$ with the gradual addition of methyl acetate solvent (different vol%).

Sample	$\tau_1(ns)$	B_1(%)	$\tau_2(ns)$	B_2(%)	$\tau_{av}(ns)$
Treated NPL	2.1	0.10 (31.2)	5.6	0.22 (68.8)	4.5
Treated NPL+ 2.4 Vol%	2.1	0.10 (31.2)	5.6	0.22 (68.8)	4.5
Treated NPL+ 4.7 Vol%	2.1	0.10 (31.2)	5.6	0.22 (68.8)	4.5
Treated NPL+ 9.0 Vol%	2.1	0.10 (31.2)	5.6	0.22 (68.8)	4.5
Treated NPL+ 13.0 Vol%	2.1	0.10 (31.2)	5.5	0.22 (68.8)	4.4
Treated NPL+ 16.7 Vol%	2.1	0.10 (31.2)	5.5	0.22 (68.8)	4.4
Treated NPL+ 20.0 Vol%	2.1	0.10 (31.2)	5.5	0.22 (68.8)	4.4

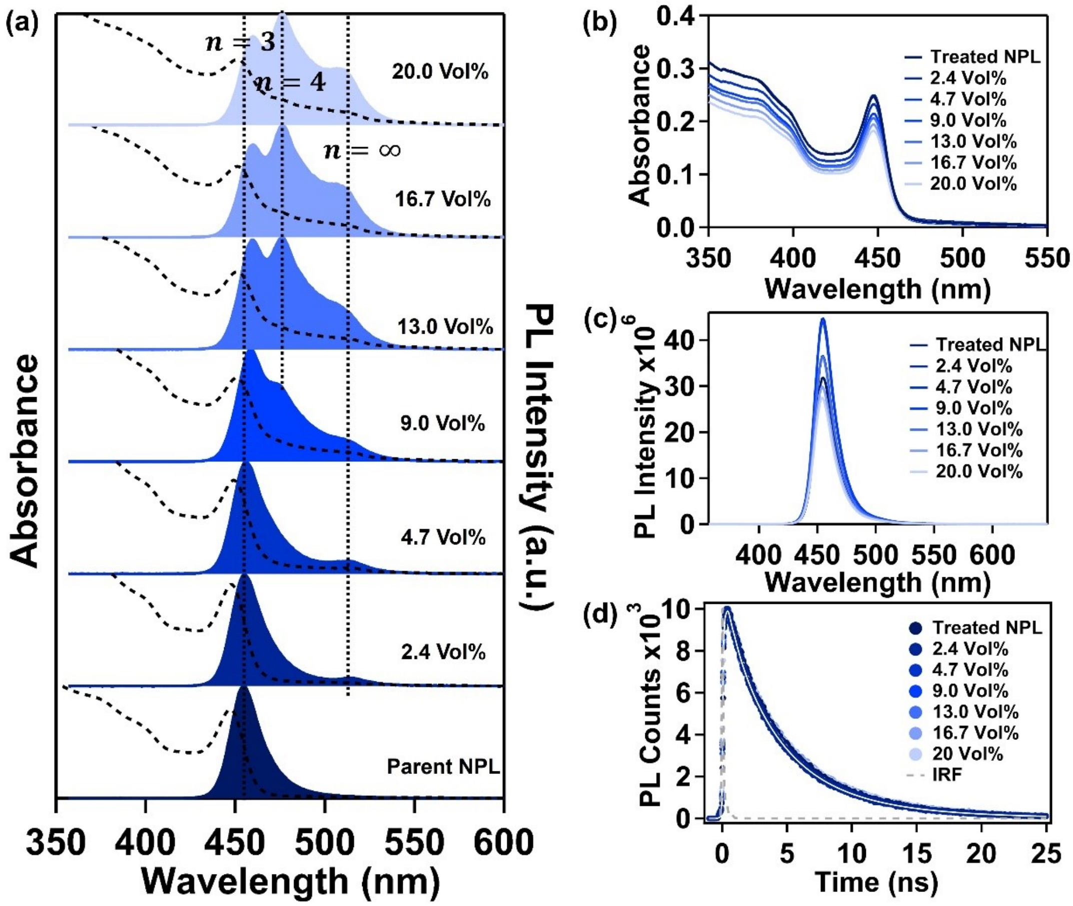

Figure S10. The comparison of stability between parent and treated NPL $(n = 3)$ against mildly polar methyl acetate solvent treatment. (a) The change in absorption and PL spectra of parent NPL with the gradual addition of methyl acetate solvent (different vol%). The change in (b) absorption spectra (c) PL spectra, and (d) PL transients of the treated NPL with the gradual addition of methyl acetate solvent (different vol%). The PL spectra were recorded by exciting the sample at 350 nm excitation. The nanosecond PL transients were recorded by exciting the sample with 375 nm pulsed diode LASER.

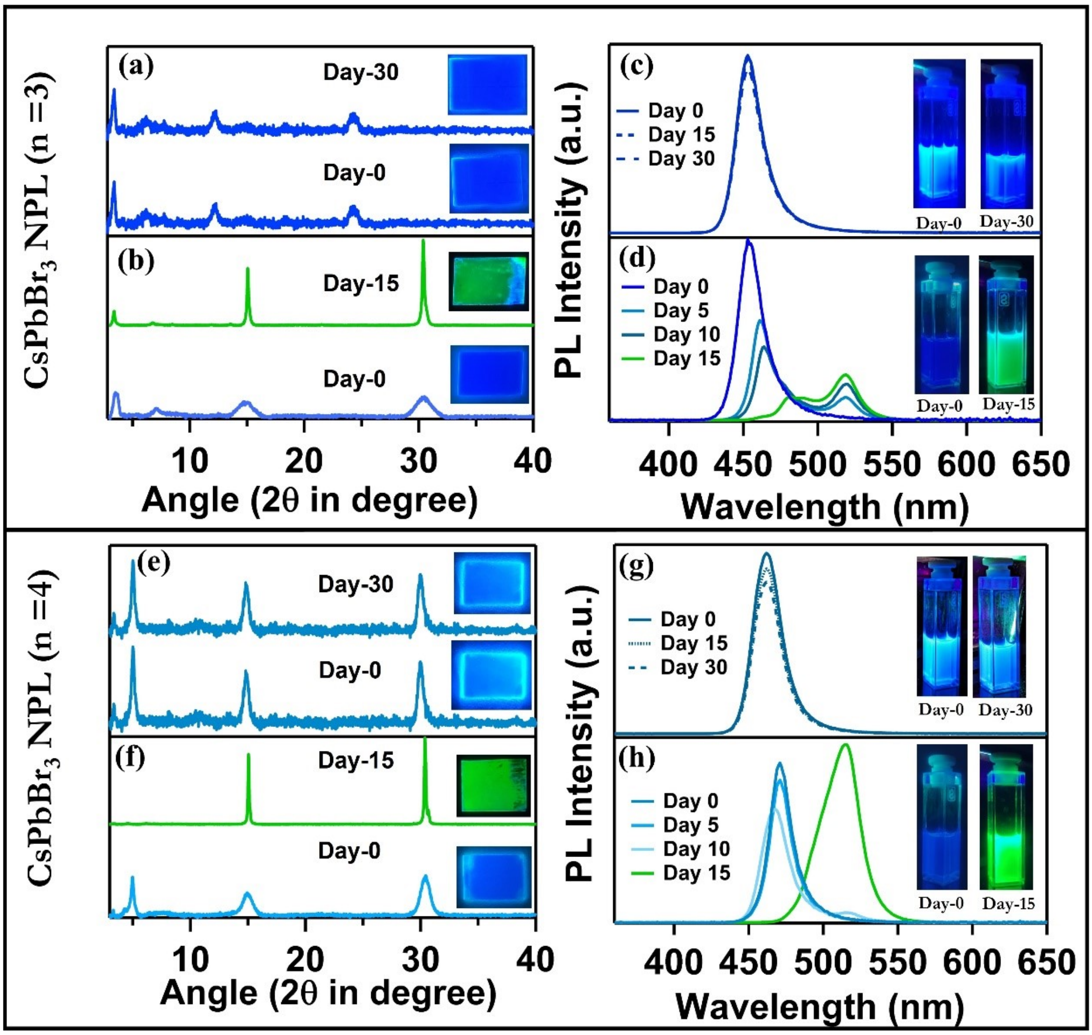

Figure S11. The ambient stability of parent and treated $CsPbBr_3$ NPL in solution (PL stability) and film (crystalline stability) stored in ambient conditions. The PXRD pattern of the (a) treated NPL ($n = 3$) at day-0 and day-30 (b) parent NPL ($n = 3$) at day-0 and day-15. The PL stability of the (c) treated NPL ($n = 3$) measured at day-0, day-15 and day-30 (d) parent NPL ($n = 3$) at day-0, day-5, day-10 and day-15. The PXRD pattern of the (e) treated NPL ($n = 4$) at day-0 and day-30 (f) parent NPL ($n = 4$) at day-0 and day-15. The PL stability of the (g) treated NPL ($n = 4$) measured at day-0, day-15, and day-30 (h) parent NPL ($n = 4$) at day-0, day-5, day-10 and day-15. The inset photographic images represent the PL of the NPL's films and colloidal solution of the parent and treated NPL captured on that mentioned day (at 365nm UV excitation).

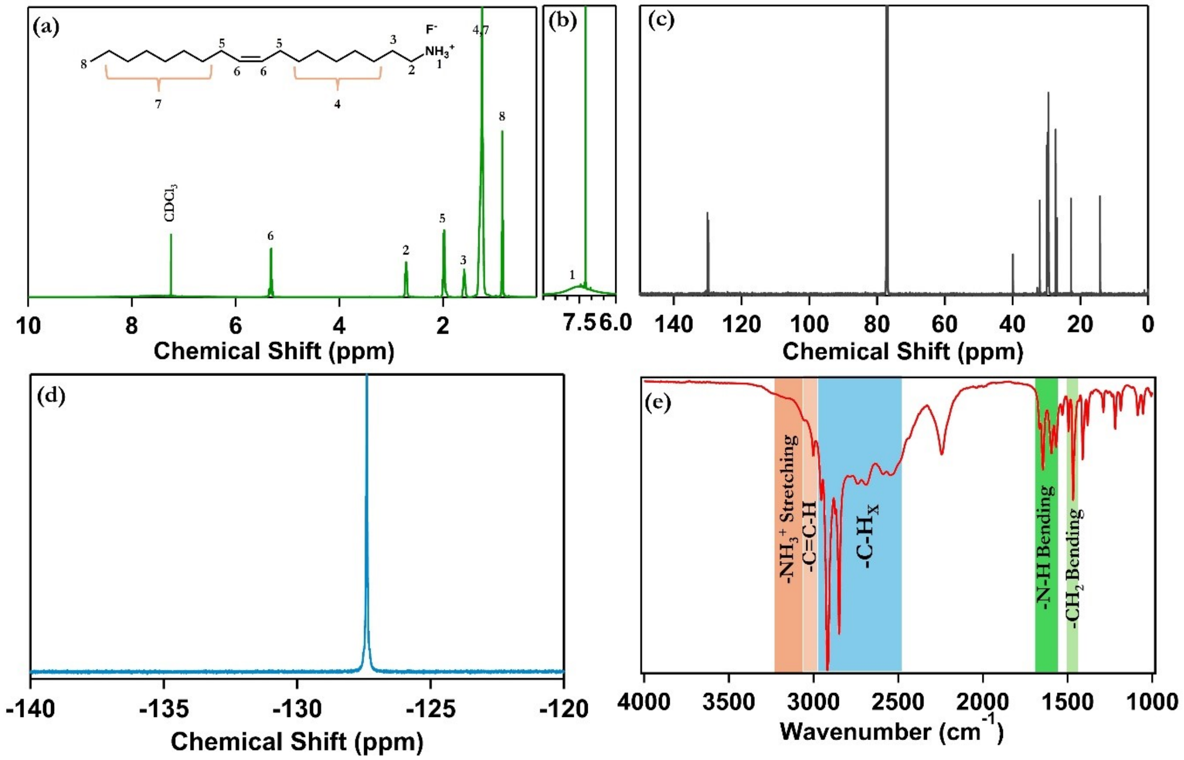

Figure S12. (a) The ^{1}H-NMR spectra of OAmF salt (b) The zoomed version of ^{1}H-NMR spectra from the figure (a) to show the presence of -NH_3^+ related proton peak. (c)^{13}C-NMR spectra of OAmF salt (d) ^{19}F-NMR spectra of OAmF salt (e) FTIR spectra of solid OAmF salt.
